# A step closer towards achieving universal health coverage: the role of gender in enrolment in health insurance in India

**DOI:** 10.1186/s12913-023-10473-z

**Published:** 2024-01-26

**Authors:** Susanne Ziegler, Swati Srivastava, Divya Parmar, Sharmishtha Basu, Nishant Jain, Manuela De Allegri

**Affiliations:** 1 Heidelberg Institute of Global Health, Medical Faculty and University Hospital, Heidelberg University, Im Neuenheimer Feld 130.3, 69120 Heidelberg, Germany; 2 Deutsche Gesellschaft für Internationale Zusammenarbeit (GIZ) GmbH, Friedrich-Ebert-Allee 32+36, 53113 Bonn, Germany; 3 Department of Population Health Sciences, School of Life Course and Population Sciences, King’s College London, Weston Education Centre, Cutcombe Road, London, SE5 9RJ United Kingdom; 4 Deutsche Gesellschaft für Internationale Zusammenarbeit (GIZ) GmbH, B5/1 Safdarjung Enclave, 110029 New Delhi, India

**Keywords:** Universal health coverage, Health insurance, Gender, India, RSBY

## Abstract

**Background:**

There is limited understanding of how universal health coverage (UHC) schemes such as publicly-funded health insurance (PFHI) benefit women as compared to men. Many of these schemes are gender-neutral in design but given the existing gender inequalities in many societies, their benefits may not be similar for women and men. We contribute to the evidence by conducting a gender analysis of the enrolment of individuals and households in India’s national PFHI scheme, *Rashtriya Swasthya Bima Yojana* (RSBY).

**Methods:**

We used data from a cross-sectional household survey on RSBY eligible families across eight Indian states and studied different outcome variables at both the individual and household levels to compare enrolment among women and men. We applied multivariate logistic regressions and controlled for several demographic and socio-economic characteristics.

**Results:**

At the individual level, the analysis revealed no substantial differences in enrolment between men and women. Only in one state were women more likely to be enrolled in RSBY than men (AOR: 2.66, 95% CI: 1.32-5.38), and this pattern was linked to their status in the household. At the household level, analyses revealed that female-headed households had a higher likelihood to be enrolled (AOR: 1.36, 95% CI: 1.14-1.62), but not necessarily to have all household members enrolled.

**Conclusion:**

Findings are surprising in light of India’s well-documented gender bias, permeating different aspects of society, and are most likely an indication of success in designing a policy that did not favour participation by men above women, by mandating spouse enrolment and securing enrolment of up to five family members. Higher enrolment rates among female-headed households are also an indication of women’s preferences for investments in health, in the context of a conducive policy environment. Further analyses are needed to examine if once enrolled, women also make use of the scheme benefits to the same extent as men do. India is called upon to capitalise on the achievements of RSBY and apply them to newer schemes such as PM-JAY.

**Supplementary Information:**

The online version contains supplementary material available at 10.1186/s12913-023-10473-z.

## Background

Universal health coverage (UHC) is set as target 3.8 of the 2030 Agenda for Sustainable Development, adopted by the member states of the United Nations in 2015. Adopting equity as its central tenet, UHC entails that everyone irrespective of socioeconomic, geographic and cultural factors has access to the quality health services they need without facing financial hardships [1]. In recent years, many low- and middle-income countries (LMICs) have started to move towards UHC by introducing publicly-funded health insurance (PFHI) schemes for poor and vulnerable populations, who usually face greater difficulties in accessing and financing health care. Systematic reviews have highlighted that enrolment into such schemes has a positive impact on the uptake of health services and on the reduction of out-of-pocket expenditure (OOPE) for health care [2, 3]. Public health insurance has been recommended as one of the most equitable means to move towards UHC [4]. Still, equity is at risk if the most vulnerable or high-risk population groups are excluded from these schemes.

This is especially true for women living in poverty and working in informal employment, as they often have no access to social health protection and avoid accessing health services due to concerns of impoverishment [1]. To reduce health risks and financial barriers for women, many countries have launched targeted programmes such as the removal of user fees and the introduction of vouchers or cash transfers for maternal or antenatal care [1, 4]. The available literature has largely examined the effect of such programmes on women. For example, the reduction and removal of user fees and the launch of conditional cash transfer programmes have increased facility-based deliveries in several LMICs [5–7].

While evidence on the effects of targeted programmes aimed at facilitating access to care and financial protection for women is increasing, there is still hardly any work being done to understand how the implementation of universal programmes ends up benefitting women as compared to men. This also applies to the implementation of PFHIs, cited earlier as a key measure to promote progress towards UHC. This paucity of evidence may be related to the assumption that a well-functioning health system moving towards UHC will automatically be equitable and gender-balanced [8], or that social health protection policies that cover entire households are per se gender-neutral and hence unbiased [9]. The limited available research focuses primarily on assessing utilisation patterns in the presence of universal social health protection initiatives, such as PFHIs. For example, health insurance membership has been found to be positively associated with women’s use of maternal health services in several LMICs [10–12]. What is lacking is an understanding of the extent to which women of all ages are actually included in emerging PFHI schemes [3]. This evidence is essential to ensure that PHFI schemes do not mirror, and hence further perpetuate, inequities that already exist at the societal level and that intended benefits are accrued by all eligible individuals, irrespective of their gender.

India is a country that has continuously been working towards UHC by introducing a number of PFHIs both at the state and at federal levels, but their gendered impact is yet to be studied in detail [8]. The *Rashtriya Swasthya Bima Yojana* (RSBY), which was launched in 2008, is an example of such a scheme. Its objective was to protect the poor from impoverishment due to OOPE for hospitalisations [13]. Although RSBY was converted into the larger *Pradhan Mantri Jan Arogya Yojana* (PM-JAY) in 2018, the scheme still presents an interesting research opportunity to better understand the role of gender in enrolment in a PFHI as the implementation arrangements of PM-JAY are similar to RSBY or other PFHIs in states [14]. RSBY policy makers progressively incorporated design features to promote the inclusion and access of women. For example, it was mandatory for spouses to be enrolled, and maternity benefits such as deliveries were included in the benefits package [13, 15]. West Bengal was the first Indian state, where women were able to enrol directly in RSBY as heads of households rather than being covered as spouses or other dependants [16]. Early research provides ambivalent results regarding women’s enrolment: women and other marginalised groups were excluded from accessing RSBY mainly because enrolment into the scheme was limited to five members per household [15, 17]. This limit also led to a preference for enrolling sons over daughters [15]. At the same time, a greater probability for enrolment in RSBY was observed for female-headed households in Maharashtra and for districts with higher numbers of female-headed households [18, 19]. In addition, initial research suggests that access to health services improved for women: once enrolled in RSBY, women were utilizing services more often than men [16, 20], but this utilisation was largely limited to women’s use of gender-specific services such as deliveries and c-sections [21]. We do not know how RSBY design features might have affected women at a later stage when RSBY was fully implemented and operated across India. Nonetheless, learnings from other Indian welfare schemes demonstrate that women’s lack of decision-making power, restricted mobility and access to resources inhibit their access to services despite being enrolled into a scheme or entitled to benefits [22–25].

A comprehensive picture of how gender affects an individual’s or a household’s probability of being enrolled or not enrolled in RSBY is lacking. Existing studies have considered a multitude of factors and are from an early stage of RSBY implementation [15], reflect the reality of one or two districts or states or only used households as units of analysis and did not explore gendered effects at the level of individuals [18–21, 26–30]. Systematic reviews on PFHIs in India that included RSBY confirmed that there is no conclusive evidence on gender differences in enrolment and utilisation, with the exception of studies reporting higher enrolment in female-headed households [9, 31].

There is an increased call at international level for the design and implementation for gender-responsive and equitable health systems, also in light of the High Level Meeting on UHC in the framework of the UN General Assembly [4, 32, 33]. There is also consensus in the international literature that gender analysis in health systems research is important, but examples how this is put in practice are lacking [34]. We aim to contribute to the international evidence base through a gender analysis of enrolment in RSBY across eight Indian states. Our objectives were to examine the role of gender in determining enrolment in RSBY. We focused on three research questions: (1) Were women in households more likely to enrol in RSBY than men and was this enrolment dependent on their age and their relationship to the head of household? (2) Were households headed by women more likely to enrol in RSBY than households headed by men?, and (3) Were female-headed households more likely to enrol either all members of a household or at least five members (in households with more than five) than male-headed households? In this paper, we used the term *sex* to describe differences arising from the biological distinction of being male or female, and *gender* to describe societal roles.

## Methods

### Study setting

Women in India’s patriarchal society suffer from high levels of inequality. India’s Gender Development Index of 0.849 in 2022 places it among the countries furthest from gender parity [35]. The inequality between men and women is especially apparent in the poor state of the public health care sector leading to poor health outcomes for women. For example, despite the successful reduction of maternal deaths, poor maternal health is still prevalent and is particularly apparent within India’s minority caste groups [36, 37]. In case essential care is not offered free of charge or in public health facilities, women often forgo care as they cannot afford private health care, which usually involves OOPE [8]. The probability that a household uses distressed financing for catastrophic hospitalization expenditure is lower for women than for men [38, 39].

PFHIs have been an essential component of India’s health care reforms and poverty reduction strategies for years [40, 41]. This includes RSBY, which at its peak covered more than 41.2 million households across India [42, 43]. The scheme provided annual insurance coverage for up to 30 000 Indian Rupees (INR) (approx. 400 EUR) per household for inpatient care for a specified list of procedures including pre-existing conditions and transportation costs of 100 INR (approx. 1.3 EUR) up to a maximum of 1000 INR (approx. 13 EUR) per hospitalisation [13]. Outpatient, preventive or primary care was not covered. Households were eligible to enrol in RSBY if they were confirmed by the Government of India to be living below the poverty line (BPL). Other poor and vulnerable population groups, such as workers of the public employment programme *Mahatma Gandhi National Rural Employment Guarantee Scheme* (MGNREGA) and similar government programmes, were included at a later stage [44]. The RSBY enrolment limit of five persons per household included the head of household, spouse and up to three dependants. Infants were automatically covered if their mothers were enrolled [13]. Initially, men were enrolled as “heads of households” in RSBY. Women were only enrolled as heads in the absence of a male head [15], but in families where the male head and spouse were both deceased, the eldest family member, preferably a woman, was listed as the new head of the household [45]. Public and private insurance companies were subcontracted for the implementation of RSBY in states. They were responsible for increasing awareness about RSBY among potential beneficiaries and for enrolling them. To access the benefits, eligible households had to enrol for RSBY i.e., all household members that were to be enrolled in the scheme had to visit the enrolment station and provide their personal information such as names, fingerprints and photos. This information was saved on a chip-based biometric insurance card that was supposed to be handed to them at the end of the enrolment process. The enrolment process had to be repeated as per the insurance premium cycle.

The decision to enrol in RSBY including the decision on which household members should be enrolled, was taken at the level of the household and usually by the male heads. This is a normal practice in India’s patriarchal society, where 85% of households are headed by men who usually also control family resources and power relations within households [46]. The enrolment decision was determined by a number of socio-economic and demographic characteristics of household members and especially the household head [26]. In this study, we focused on gender as a driver for enrolment. In light of India’s overall gender bias, we considered the following hypotheses: first, women enjoyed a lower likelihood of being enrolled in RSBY, but this effect might have been attenuated by their age and their positions within a household, i.e., whether they were a head of the household, spouse, daughter or other female household members. Second, households headed by women were, on the one hand, more likely to enrol and, one the other hand, to enrol as many household members as possible as a result of women’s greater health risk aversion.

### Data sources and sampling

This study uses data from a cross-sectional household survey conducted in 2014 in Bihar, Gujarat, Kerala, Mizoram, Tripura, Uttarakhand, Uttar Pradesh and West Bengal [16]. The objective of the survey was to examine whether RSBY had improved access to health care and reduced OOPE for beneficiaries who accessed health services under the scheme, but given rich data collection, the survey enabled the pursuit of multiple research questions.

**Fig. 1:**
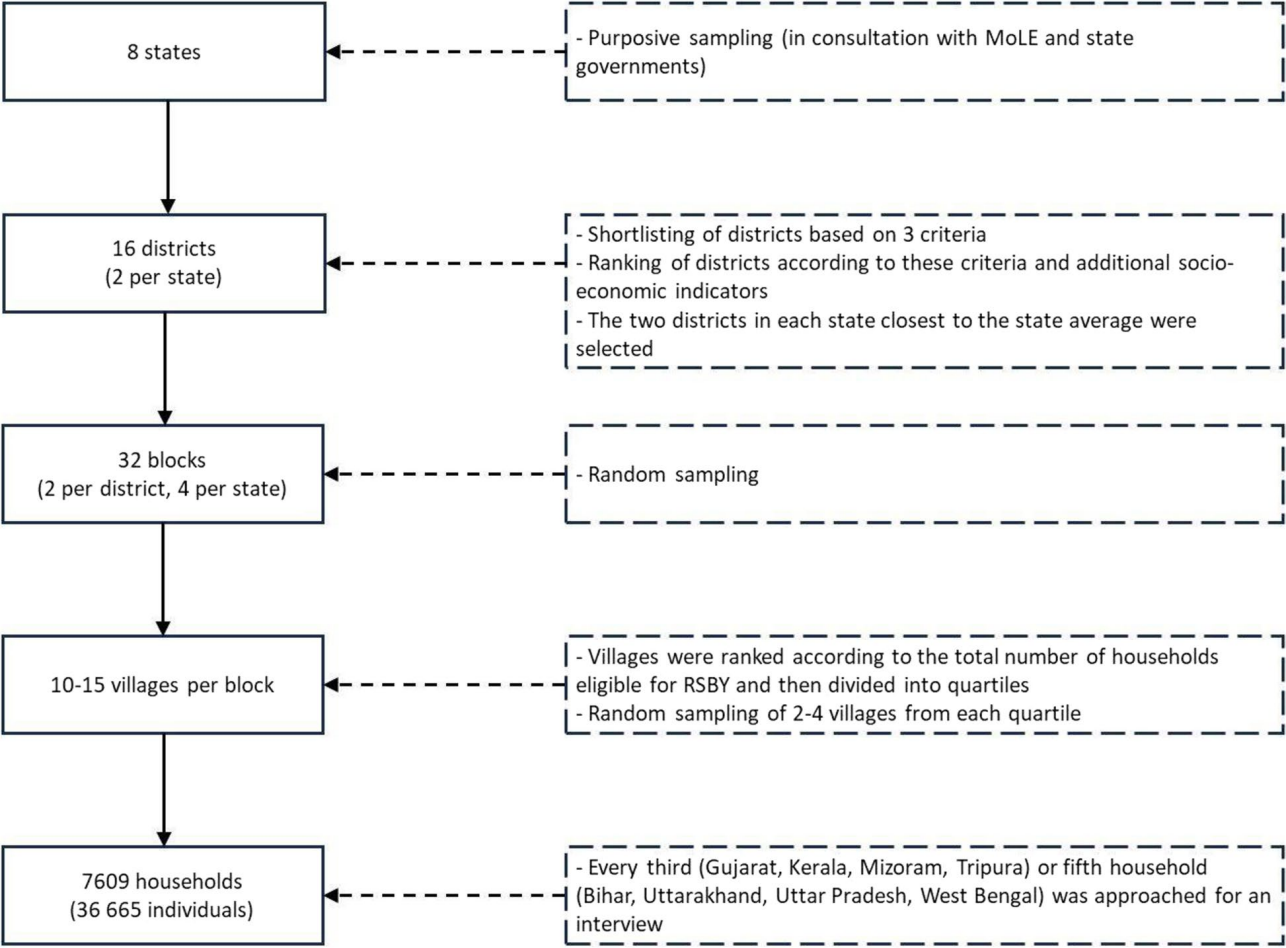
Flowchart of the multi-stage purposive and random sampling strategy. Source: own depiction. Abbreviation: MoLE = Ministry of Labour and Employment

The study population included households that were eligible for RSBY. This meant they were confirmed as BPL, were registered for MGNREGA, the subsidised food programme *Antyodaya Anna Yojana* or had an RSBY card in the past. Study participants were drawn using a multi-stage purposive and random sampling technique. The eight states were purposively selected in consultation with the Indian central and state governments. The states were chosen to reflect the demographic and socio-economic diversity of Indian states, as well as the varying progress of RSBY implementation across states. From each state, districts were shortlisted based on three criteria: an enrolment rate of RSBY beneficiaries between 50% and 60%, a hospitalisation rate above 3% (meaning that among enrolled beneficiaries at least 3% were hospitalized) and at least three years of RSBY implementation. These criteria were established based on the research questions of the original survey, which aimed to examine various aspects such as RSBY enrolment and utilization during hospitalization. Consequently, data were collected from districts with high rates of RSBY enrolment and hospitalization. The inclusion of three years of RSBY implementation ensured that the scheme was fully operational and not in its initial stages. Districts were then ranked based on these criteria and additional socio-economic indicators. The two districts most similar to the state average were selected. In each district, two blocks (administrative sub-divisions of a district in rural India) were randomly selected. The sample size for each district was distributed across the two blocks in the ratio of the actual numbers of households eligible for RSBY in each block. Villages in these blocks were ranked according to the total number of eligible households and afterwards divided into quartiles. From each quartile, two to four villages were randomly selected, which led to the selection of 10 to 15 villages in each block. In each selected village, every third (for Gujarat, Kerala, Mizoram, Tripura) or fifth (for Bihar, Uttarakhand, Uttar Pradesh, West Bengal) household was approached for the survey using the right thumb rule. This led to approx. 1000 interviewed households each for Gujarat, Kerala, Mizoram, and Tripura and 900 each for Bihar, Uttarakhand, Uttar Pradesh and West Bengal. The main respondent for the survey was generally the head of the household. In case the head of household was absent, the spouse was interviewed. In the absence of the spouse, other senior household members such as the father or mother of the head of household were interviewed. Figure 1 depicts the sampling strategy.

The survey consisted of a pre-tested paper-based questionnaire that was translated into the relevant local languages and administered by trained interviewers fluent in these languages. The questionnaire covered topics related to health insurance literacy, awareness and knowledge about RSBY, access to health care, utilisation of health services and health care expenditure. For this paper, we analysed the survey modules on socio-economic and demographic characteristics of households and individuals and their enrolment in RSBY. We dropped families with a missing RSBY enrolment status for all members (*n*=12) from the analysis. This led to a study sample of 7609 households and 36 665 individuals across all eight states.

### Variables and their measurement

Based on the three research questions, the analysis took into consideration three binary outcome variables, namely individual enrolment, household enrolment and complete household enrolment. As the objective of the analysis was to understand gender as both an individual and household driver for enrolment, therefore, sex (male or female) was the key exposure variable. To account for potential confounders, we included a set of covariates that captured socio-economic, demographic and geographic characteristics of individuals and households. Table 1 provides an overview of the outcome variables, the main exposure variable and covariates, as well as their measurement and distribution in the sample.

**Table 1:** Used variables, their measurement and their distribution in the sample (in total and disaggregated by sex)

**Variables**	**Measurement**	**Distribution**					
		**Total**		**Men**		**Women**	
		n	%	n	%	n	%
**Outcome variables**							
individuals enrolment in RSBY	0 = no	18847	51.66	9766	51.82	9081	48.18
	1 = yes	17638	48.34	9019	51.13	8619	48.87
	*missing*	*180*					
households enrolment in RSBY	0 = no	3170	41.66	2937	92.65	233	7.35
	1 = yes	4439	58.34	3829	86.26	610	13.74
households completely enrolled in RSBY	0 = no	806	18.16	711	88.21	95	11.79
	1 = yes	3633	81.84	3118	85.82	515	14.18
**Main exposure variable**							
sex	1 = male	18877	51.49	-		-	
	2 = female	17788	48.51	-		-	
**Covariates individual level**							
age	1 = 0-14	10312	28.12		51.49		48.51
	2 = 15-49	19996	54.54		51.38		48.62
	3 = 50+	6357	17.34		51.82		48.18
relationship to hoh	1 = head of household	7609	20.75		88.92		11.08
	2 = spouse	6281	17.13		1.80		98.20
	3 = child	16709	45.57		58.31		41.69
	4 = others	6066	16.54		37.17		62.83
education	1 = no education	12154	33.15		43.36		56.64
	2 = up to 7 years	12921	35.24		53.54		46.46
	3 = 8 years and above	11590	31.61		57.71		42.29
occupation	1 = farming	4794	13.08		81.50		18.50
	2 = labourer/ daily wages	5436	14.83		84.44		15.56
	3 = other occupation	2266	6.18		78.11		21.89
	4 = student	10350	28.23		53.04		46.96
	5 = housewife	8136	22.19		2.94		97.06
	6 = not employed	3105	8.47		50.60		49.40
	7 = not yet in school	2578	7.03		50.81		49.19
**Covariates household level**							
caste	1 = no caste/ other	7168	19.62		50.70		49.30
	2 = SC+ST	15969	43.70		51.41		48.59
	3 = OBC	13404	36.68		51.99		48.01
	*missing*	*124*					
religion	1 = hindu	24556	66.97		52.17		47.83
	2 = muslim	6586	17.96		50.47		49.53
	3 = christian/ other	5523	15.06		49.67		50.33
BPL status	1 = BPL	23956	65.34		51.68		48.32
household size (mean and SD)	continuous	5.573	2.078		2.076		2.081
wealth index	1 = 1st quintile = poorest	6542	17.84		50.78		49.22
	2 = 2nd quintile	7370	20.10		51.79		48.21
	3 = 3rd quintile	7513	20.49		50.99		49.01
	4 = 4th quintile	7398	20.18		51.88		48.12
	5 = 5th quintile = least poor	7842	21.39		51.89		48.11
state	1 = Bihar	4855	13.24		53.16		46.84
	2 = Uttarakhand	4593	12.53		52.34		47.66
	3 = Uttar Pradesh	5322	14.52		52.82		47.18
	4 = West Bengal	3573	9.74		51.55		48.45
	5 = Gujarat	5167	14.09		52.06		47.94
	6 = Kerala	4287	11.69		47.42		52.58
	7 = Mizoram	4468	12.19		49.75		50.25
	8 = Tripura	4400	12.00		52.11		47.89

Missing values were automatically dropped from the analysis. Absolute numbers are reported for the sample size, outcome and key exposure variables

*Abbreviations*: *BPL *Below poverty line, *RSBY* Rashtriya Swasthya Bima Yojana, *hoh *Head of household, *SC *Scheduled caste, *ST *Scheduled tribe, *OBC *Other backward class, *SD *Standard deviation

### Analytical approach

The three outcome variables were constructed as follows: (1) individual enrolment was constructed for every member of a household and was based on the question whether an individual household member was enrolled or not enrolled in RSBY; (2) household enrolment was constructed for every household with all its members as a single unit of analysis. A household was categorised as enrolled if at least one household member was enrolled in RSBY; (3) complete household enrolment was also constructed with households as units of analysis. A household was considered as completely enrolled if all its members or at least five members in households with more than five members were enrolled in RSBY.

We initially used descriptive statistics to illustrate the distribution of the outcome variables and to identify how many individuals and households were enrolled in RSBY. We then conducted bivariate analyses (see Supplementary file, Tables S1, S2 and S3) to explore the distribution of the covariates among enrolled and non-enrolled individuals and households to inform the subsequent models. We conducted multivariate logistic regressions using Stata 16.1 [47] to assess the association between the three outcome variables and sex while controlling for socio-economic and demographic characteristics of individuals and households and, dependent on the model, adjusted for clustering at household and district levels. For each outcome variable, we ran the regression twice: first on the pooled sample, which included data of all eight states, and second on each state to examine differences between states. In addition, we carried out sensitivity analyses (see Supplementary file, Tables S4 and S5) to test if the results observed were robust in light of the model underlying assumption. The regression models were constructed as follows:

#### Outcome 1: individual enrolment

We measured the likelihood of an individual, situated within a given household, being enrolled. The equation for estimating individual enrolment was:

1 $${RSBY}_{ind} = {\upbeta }_{0} + {\upbeta }_{sexind} + {\upbeta }_{covind} + {\upbeta }_{covhh} + {\upbeta }_{sex*age} + {\upbeta }_{sex*relationship} + ({\upbeta }_{state}) +\upvarepsilon$$

*RSBY*_*ind*_ was the binary outcome variable taking the value ‘1’ for enrolment of an individual and ‘0’ for non-enrolment. *sexind* was the main exposure variable. *covind* included the socio-economic characteristics of individuals, namely age, relationship to the head of household, education and occupation. *covhh* referred to the socio-economic characteristics of households and included caste, religion, BPL status, household size and wealth index. The geographic location *state* was only used for the pooled sample. We clustered at the household level, as individuals living in the same household share similar characteristics.

As we assumed that the decision to enrol a woman in RSBY was dependent on her age and on her position within a household, we added selected interaction terms to the model to understand if and to what extent the covariates age (sex*age) and relationship to the head of household (sex*relationship) mediated the effect of sex on the enrolment of individuals. We applied the Wald test to test variable inclusion and used the Likelihood Ratio Test (LRT) to test for interactions and final model specification. The Wald test confirmed (*p* < 0.001) that including the chosen variables improved the statistical fit of the model. The LRT involved the testing of two models, the first model without the interactions and the second with the interactions. The test confirmed (*p* = 0.0837) that the first model is nested in the second meaning that the inclusion of the interactions statistically improved the final model fit.

#### Outcome 2: household enrolment

Household enrolment measured the likelihood of a female-headed household being enrolled or not being enrolled in RSBY. We carried out the analysis among all households of the sample (*n*=7609), but only considered characteristics of the head of household. The enrolment status of a household was written as:

2 $${RSBY}_{hh} = {\upbeta }_{0} + {\upbeta }_{sexhoh} + {\upbeta }_{covhoh} + {\upbeta }_{covhh} + ({\upbeta }_{state}) +\upvarepsilon$$

Most of the variables were the same as in (1), with the difference that the covariate *relationship to the head of household* became obsolete, and interaction terms were not used since they were no longer conceptually relevant. We clustered effects at the district level.

#### Outcome 3: complete household enrolment

Complete household enrolment measured the likelihood of female-headed households enrolling all members of a household or at least five in households with more than five members. We used the same model as in (2) including clustering at the district level, but applied it to a sub-sample that only included enrolled households (*n*=4439).

## Results

To keep the focus on gender, we report the results for each model, focusing exclusively on the exposure of interest and preceded by a brief description of sample characteristics. Complete model results are reported in Supplementary file, Tables S6, S7 and S8.

### Outcome 1: individual enrolment

Out of the total sample of 36 665 individuals, 51.5% were men and 48.5% were women. A total of 48.3% of individuals reported enrolment out of which 51.1% were men and 48.9% were women. The chi-squared test showed no significant difference in the distribution of the outcome variable and the main exposure variable sex, neither for the pooled sample nor for any of the study states (see Supplementary file, Table S1).

Results from the multivariate logistic regression for the pooled sample shown in Table 2 revealed that, even while adjusting for all covariates, women had a statistically significant higher probability of being enrolled than men (adjusted odds ratio, AOR: 1.27, 95% confidence interval, CI: 1.003-1.6). A woman’s age did not mediate the relationship between sex and enrolment, while her status within the household did. We observed that daughters (AOR: 0.76, 95% CI: 0.61-0.95) and other female household members (AOR: 0.8, 95% CI: 0.64-0.998) had a lower likelihood to be enrolled than male household members and female spouses.

**Table 2:** Results of the multivariate logistic regression for individual enrolment (outcome 1)

	**Pooled**							**Bihar**						**Uttarakhand**						**Uttar Pradesh**					**West Bengal**			
	AOR		95% CI			p		AOR	95% CI			p		AOR		95% CI			p	AOR	95% CI		p		AOR	95% CI		p
sex ind																												
male	1							1						1						1					1			
female	1.265		1.003		1.595	0.048		1.289	0.670		2.470	0.445		2.664		1.315		5.397	0.007	0.793	0.354	1.772	0.571		0.964	0.359	2.591	0.943
sex # age categories																												
female # 15-49	0.996		0.870		1.140	0.952		1.178	0.800		1.720	0.400		0.685		0.448		1.047	0.080	1.044	0.712	1.531	0.825		1.099	0.651	1.857	0.723
female # 50+	1.018		0.864		1.200	0.832		1.082	0.690		1.690	0.728		0.586		0.350		0.981	0.042	1.052	0.651	1.698	0.837		1.096	0.576	2.086	0.781
sex # relationship																												
female # spouse	0.930		0.582		1.485	0.761		0.638	0.170		2.360	0.501		0.357		0.085		1.497	0.159	2.747	0.555	13.583	0.215		1.642	0.395	6.833	0.495
female # child	0.763		0.614		0.949	0.015		0.642	0.340		1.200	0.166		0.546		0.286		1.042	0.067	1.228	0.574	2.628	0.596		0.867	0.333	2.257	0.769
female # others	0.800		0.642		0.998	0.048		0.718	0.380		1.360	0.312		0.530		0.253		1.111	0.093	1.009	0.468	2.174	0.982		0.540	0.212	1.378	0.197
	**Gujarat**							**Kerala**						**Mizoram**						**Tripura**								
	AOR	95% CI					p	AOR	95% CI				p	AOR	95% CI				p	AOR	95% CI			p				
sex ind																												
male	1							1						1						1								
female	0.990	0.484		2.027			0.978	1.246	0.648	2.399			0.510	0.978	0.496		1.929		0.950	1.146	0.547	2.400		0.717				
sex # age categories																												
female # 15-49	0.801	0.555		1.157			0.237	1.022	0.652	1.604			0.920	1.178	0.798		1.740		0.409	0.836	0.562	1.244		0.377				
female # 50+	1.045	0.676		1.614			0.844	1.021	0.602	1.731			0.940	1.285	0.780		2.116		0.325	0.786	0.439	1.409		0.419				
sex # relationship																												
female # spouse	1.126	0.339		3.741			0.846	1.982	0.319	12.332			0.460	1						1.543	0.383	6.211		0.542				
female # child	1.047	0.534		2.056			0.893	0.691	0.374	1.279			0.240	0.963	0.505		1.836		0.909	0.867	0.435	1.730		0.685				
female # others	1.039	0.545		1.979			0.908	0.721	0.386	1.347			0.310	1.406	0.702		2.817		0.336	1.091	0.516	2.306		0.819				

This table only shows the results for sex, they key variable of interest. Full model results are shown in the Supplementary file, Table S6

Baselines for the interaction *sex* and *age categories*: male#0-14, male#15-49, male#50+, female#0-14;

Baselines for the interaction *sex* and *relationship to the head of household*: male#hoh, male#spouse, male#child, male#others, female#hoh

*Abbreviations*: *ind *Individual, *AOR *Adjusted odds ratio, *CI *Confidence interval, *p* *p*-value

The state-specific analysis revealed that women in Uttarakhand had a higher probability to be enrolled in RSBY than men (AOR: 2.66, 95% CI: 1.32-5.38), but women older than 50 years were less likely to be enrolled (AOR: 0.59, 95% CI: 0.35-0.98), adjusting for all variables. We did not observe such effects for the other study states.

Based on these results, we carried out a sensitivity analysis to understand if the state of Uttarakhand drives the overall results of the pooled sample (see Supplementary file, Table S4). By deleting Uttarakhand from the pooled sample, we observed no effect between sex and enrolment in RSBY (AOR: 1.18, 95% CI: 0.92-1.5).

### Outcome 2: household enrolment

The sample included 7609 households out of which 58.3% were enrolled in RSBY. A total of 86.3% of the enrolled households were headed by men and 13.7% by women. Supplementary file, Table S2, describes the bivariate results. The chi-squared test revealed significant differences between the outcome variable and sex for the pooled sample and for the states of Uttarakhand, West Bengal and Mizoram.

Findings of the multivariate logistic regression shown in Table 3 indicate that, adjusting for all covariates, households headed by women had a statistically significant higher probability of being enrolled in RSBY than households headed by men (AOR: 1.36, 95% CI: 1.14-1.62).

**Table 3:** Results of the multivariate logistic regression for household enrolment (outcome 2)

	**Pooled**				**Bihar**				**Uttarakhand**				**Uttar Pradesh**				**West Bengal**			
	AOR	95% CI		p	AOR	95% CI		p	AOR	95% CI		p	AOR	95% CI		p	AOR	95% CI		p
sex hoh																				
male	1				1				1				1				1			
female	1.358	1.142	1.616	0.001	1.460	0.968	2.202	0.071	2.507	1.142	5.503	0.022	0.862	0.813	0.914	0.000	1.278	0.772	2.116	0.340
	**Gujarat**				**Kerala**				**Mizoram**				**Tripura**							
	AOR	95% CI		p	AOR	95% CI		p	AOR	95% CI		p	AOR	95% CI		p				
sex hoh																				
male	1				1				1				1							
female	0.958	0.705	1.302	0.783	1.056	0.791	1.409	0.712	1.652	0.955	2.857	0.072	1.126	0.493	2.568	0.778				

This table only shows the results for sex, they key variable of interest. Full model results are shown in the Supplementary file, Table S7

*Abbreviations*: *hoh *Head of household, *AOR *Adjusted odds ratio, *CI *Confidence interval, *p p*-value

Looking at states, we noted similar significant results for female-headed households in Uttarakhand (AOR: 2.51, 95% CI: 1.14-5.5). Also, in Bihar and Mizoram, female-headed households had 1.46 and 1.65 the odds of being enrolled than male-headed households, respectively, although the results were insignificant at the 95% confidence interval. In Uttar Pradesh, female-headed households were less likely to enrol in RSBY than male-headed households (AOR: 0.86, 95% CI: 0.81-0.91). We did not find a significant association between the sex of the head of a household and enrolment in the other states.

We carried out a sensitivity analysis by dropping Uttarakhand from the pooled sample (Supplementary file, Table S5) and observed that female-headed households were still more likely to enrol in RSBY than male-headed households (AOR: 1.23, 95% CI: 1.11-1.49).

### Outcome 3: complete household enrolment

Complete household enrolment was examined among households that were enrolled in the first place. This was the case for 4439 households and out of these, 81.8% reported complete enrolment. A total of 85.8% of households with complete enrolment were headed by men and 14.2% by women. The chi-squared tests in Supplementary file, Table S3, showed a significant difference in the distribution of the outcome variable and sex for the pooled sample, but not for any of the study states.

The results of the multivariate logistic regression are shown in Table 4. We observed that complete enrolment of a household in RSBY was not dependent on whether the household was headed by a man or a woman.

**Table 4:** Results of the multivariate logistic regression for complete household enrolment (outcome 3)

	**Pooled**				**Bihar**				**Uttarakhand**				**Uttar Pradesh**				**West Bengal**			
	AOR	95% CI		p	AOR	95% CI		p	AOR	95% CI		p	AOR	95% CI		p	AOR	95% CI		p
sex hoh																				
male	1				1				1				1				1			
female	0.826	0.603	1.130	0.232	0.893	0.120	6.640	0.912	0.419	0.117	1.505	0.183	1.739	0.145	20.916	0.663	0.700	0.297	1.652	0.415
	**Gujarat**				**Kerala**				**Mizoram**				**Tripura**							
	AOR	95% CI		p	AOR	95% CI		p	AOR	95% CI		p	AOR	95% CI		p				
sex hoh																				
male	1				1				1				1							
female	0.803	0.500	1.289	0.363	0.626	0.300	1.308	0.213	0.704	0.178	2.784	0.617	1.049	0.907	1.212	0.522				

This table only shows the results for sex, they key variable of interest. Full model results are shown in the Supplementary file, Table S8

*Abbreviations*: *hoh *Head of household, *AOR *Adjusted odds ratio, *CI *Confidence interval, *p p*-value

## Discussion

This study makes an important contribution to the literature by providing a first detailed assessment of the role gender plays in shaping decisions to enrol in RSBY, a nation-wide PFHI launched in India to foster progress towards UHC. Although the scheme has now been replaced by PM-JAY, our study is still highly relevant for India and similar settings, since it advances our understanding of how gender can determine participation in universal schemes. Three key lessons emerge from our findings: first, albeit at first glance it appears that all women might have enjoyed greater chances of being enrolled in RSBY, it is in fact their position within a household that was decisive in determining whether or not they were enrolled. Second, female-headed households enjoyed a greater probability of being enrolled, and third, they did not necessarily achieve complete enrolment. Hereafter, we examine each of these findings and appraise them in relation to prior literature on RSBY and the wider Indian socio-political and cultural context.

First, we note that while the analysis on the pooled sample on the enrolment of individuals suggested that women were more likely to be enrolled, analysis at the state level revealed large heterogeneity, with no difference in the enrolment of men and women in most states except Uttarakhand. Even after dropping Uttarakhand, we observed no difference. This means that overall enrolment in RSBY was largely gender-neutral, a result that is probably attributable to the mandatory enrolment of spouses. Nonetheless, for a country like India where gender inequality in health care is well-documented [38, 39, 48–53], this is a very encouraging result.

Taking a closer look at states, we note that a higher enrolment of women in Uttarakhand was already observed in the early years of RSBY implementation [54]. The results are difficult to explain, but this might be linked either to a migration of men to urban areas leaving women in charge of families and agricultural production [55], or to the efforts undertaken by insurance companies to enrol beneficiaries that varied across states and districts as a result of differences in governance of implementation [56]. In order to better understand the results for Uttarakhand, additional research is required.

Further analysis shows that despite RSBY observed gender-neutrality in enrolment, RSBY was not necessarily pro-women as the enrolment of women was largely linked to their relationship to the household head, with spouses being more likely to be enrolled, but not daughters or other female household members. Again, in line with what is mentioned earlier, this pattern is probably attributable to the design structure of the scheme, whereby it was mandatory to enrol spouses. In Uttarakhand, older women were less likely to enrol than younger women. We note that such structural limitations on the maximum number of household members to be enrolled may be dictated by cost considerations, inevitable in the design of health insurance schemes in LMICs. We urge policy makers to lift such limits over time in order to allow the scheme to progress towards universalism. Based on learnings from RSBY, India’s PM-JAY does not have a limit on the number of household members anymore [57].

Second, our findings from the pooled sample indicate that female-headed households had a 36% higher likelihood to be enrolled in RSBY than male-headed households. Less heterogeneity was observed for this outcome than for individual-level enrolment, a finding confirmed also by our sensitivity analysis (see Supplementary file, Table S5) and by previous studies on RSBY [18, 19]. Our results are encouraging as female-headed households in India are generally considered more vulnerable and possess fewer assets than male-headed households [58, 59]. Nonetheless, they have greater autonomy in terms of taking decisions [60] which might have resulted in higher RSBY enrolment rates of female-headed households. Another explanation for our results might be related to the RSBY guidelines as it was mandatory for the household head to be physically present during the enrolment process. Male household heads might have been at work or migrated to other states or cities for economic purposes which increased the chances for the enrolment of women as heads of households [19]. Our findings are well aligned with the international literature, which documents that women tend to invest substantially more in the health care of their family ahead of time. For example, a study in Ethiopia identified female-headed households as significantly more likely to enrol in CBHI than male-headed households [61]. In Nepal, women from female-headed households were more likely to use health services than women from male-headed households [62], and they were less likely to experience child death [63].

The results are not uniform across India, and certain states and regions require additional focus. In Uttar Pradesh we observed that female-headed households were less likely to be enrolled in RSBY than male-headed ones. This result is not surprising, as women in India’s most populous state have repeatedly been identified as particularly vulnerable. For example, sex and maternal mortality ratios as well as female literacy and workforce participation rates in Uttar Pradesh are amongst the worst in all of India [64–67].

The fact that female heads of households were such an important driver for the enrolment of a household in RSBY should be considered by PM-JAY policy makers and implementers. To some extent, this is already the case as PM-JAY insurance cards are now issued for every enrolled member of a household. Important implications for future PFHI designs include, for example, enrolling women as primary beneficiaries not only in the absence of a male head, but as equals and promoting awareness campaigns targeted specifically at women encouraging them to enrol even when men decide not to do so. We also recommend additional research regarding the impact that female-headed households can have on the uptake and utilisation of health insurance.

Third, our analysis revealed that achieving complete enrolment in RSBY, i.e., when all members of the household are enrolled, was not dependent on whether a household was headed by a man or a woman. This finding may initially appear surprising considering the fact that enrolment was higher among female-headed households. Nonetheless, it needs to be appraised against the fact that out of all enrolled households, only 82% of households were completely enrolled. This means that 18% of enrolled households were enrolling fewer than the five members stipulated by the scheme policy. Although women were more likely to enrol households in RSBY, they did not have more means than men to overcome the structural barriers of the scheme such as perverse incentives of insurance companies or the lack of awareness among beneficiaries about the functioning of the scheme. For instance, insurance companies received premium payments per BPL household enrolled, and not per individual enrolled. This motivated companies to enrol as many households as possible, but did not provide an incentive to enrol as many individuals allowed per household [15, 30]. In the early years of RSBY, insurance companies were also responsible for raising awareness and knowledge levels about the scheme among potential beneficiaries, but these levels remained low throughout the implementation of RSBY even among enrolled beneficiaries [27, 29]. Higher awareness and knowledge levels, especially among women, might have led to higher enrolment and utilisation rates. This would have resulted in higher insurance claims and consequently lower profits for insurance companies [13]. Although this ambiguity was already known in the early years of the implementation of RSBY, it was never changed. We urge policy makers to regulate key implementers tasked with the implementation of PFHI to avoid such perverse incentives.

### Methodological considerations

This is the only paper that focuses on women’s enrolment in RSBY across eight Indian states. Despite this strength, we need to acknowledge the following limitations: first, the purposive selection of districts with high enrolment rates was a result of the initial objective of the survey. This selection might have affected the distribution of enrolment in a non-random way. Second, the efforts undertaken by insurance companies to enrol beneficiaries in RSBY varied across states and districts. This might explain differences in the enrolment rates in states and districts. Both limitations were beyond the purview of our data source and did not affect the results of this study, as we did not analyse overall RSBY enrolment rates.

The results of this paper may not be generalised to settings that are different from the study states and districts that were selected for the household survey we analysed. Additional qualitative research might help to understand what causes the observed effects. Findings should be further validated by larger studies in India and other LMICs. Furthermore, as this paper focuses on enrolment in PFHI, we recommend research that examines utilization and financial protection of women and men having access to universal schemes versus targeted schemes.

## Conclusion

Our findings deliver important contributions to the following evidence base: first, in settings where women are confronted with high levels of vulnerability and exclusion, health insurance schemes need to be designed and implemented in a gender-responsive and equitable way. Otherwise, such schemes will mirror patterns of exclusion or inequities that exist at a societal level [4]. This entails that policy makers need to ensure with the onset of a health insurance scheme that it is not characterised by technical or structural design features that lead to the systematic exclusion of women and girls. This can be avoided by including women and people from vulnerable population groups in leadership and governance regarding the design and implementation of health insurance schemes, and by applying a gender lens at all levels of implementation of a PFHI, starting with gender-sensitive awareness and enrolment campaigns to ensure that women and men can access health services equally.

Second, female-headed households play a decisive role in securing access to health insurance. Exposure to female leaders has also helped to reduce gender gaps in health care utilisation [68]. The role of women in leadership positions regarding health care access and health seeking behaviour is an under-researched item. There is also a need for building an evidence base around women and the opportunities and obstacles they face while exercising their rights within UHC and health systems reforms. The sooner this evidence can be built, the sooner specific measures and strategies that target barriers to health care access for women and girls can be integrated into the design of health programmes.

PM-JAY is India’s largest step towards achieving UHC, but it has not yet managed to reduce gender disparities despite its universal approach. For example, the fact that all members of a household have to be physically present for the verification process or that women are less aware about the scheme could lead to women being left out [69]. We conclude by calling on PM-JAY policy makers and implementers to urgently integrate a gender-sensitive and equitable design into the already existing scheme and adopt measures that specifically target women and girls. Otherwise, India’s inequalities at the societal level will continue to reflect in PM-JAY, making equity in access to health services and the achievement of UHC more challenging.

### Supplementary Information

**Additional file 1: Table S1.** Results of the bivariate analysis for individual enrolment (outcome 1). **Table S2.** Results of the bivariate analysis for household enrolment (outcome 2). **Table S3.** Results of the bivariate analysis for complete household enrolment (outcome 3). **Table S4.** Sensitivity analysis for individual enrolment (outcome 1). **Table S5.** Sensitivity analysis for household enrolment (outcome 2). **Table S6.** Results of the multivariate logistic regression for individual enrolment (outcome 1). **Table S7.** Results of the multivariate logistic regression for household enrolment (outcome 2). **Table S8.** Results of the multivariate logistic regression for complete household enrolment (outcome 3).

## Data Availability

The original data are available with the Deutsche Gesellschaft für Internationale Zusammenarbeit (GIZ) GmbH in New Delhi, India. GIZ carried out the original data collection on behalf of the Indian Government (Ministry of Labour and Employment). The data are not publicly available. The authors obtained written permission from GIZ to use the data for the purpose of this study and GIZ shared the data with the authors in fully anonymised format. Data may be shared on request to the corresponding author with permission of GIZ.

## Abbreviations

AOR: Adjusted odds ratio
BPL: Below poverty line
CI: Confidence interval
INR: Indian Rupee
LMIC: Low and middle income country
MNGREGA: Mahatma Gandhi National Rural Employment Guarantee Scheme
OOPE: Out-of-pocket expenditure
PFHI: Publicly-funded health insurance
PM-JAY: Pradhan Mantri Jan Arogya Yojana
RSBY: Rashtriya Swasthya Bima Yojana
UHC: Universal Health Coverage
