# Supplementary material for: A step closer towards achieving universal health coverage: the role of gender in enrolment in health insurance in India
Source: BMC Health Serv Res. 2024 Jan 26;24:141. doi: 10.1186/s12913-023-10473-z (PMC10821565; doi:10.1186/s12913-023-10473-z)
Supplement: Supplementary file 1 — Additional file 1: Table S1. Results of the bivariate analysis for individual enrolment (outcome 1). Table S2. Results of the bivariate analysis for household enrolment (outcome 2). Table S3. Results of the bivariate analysis for complete household enrolment (outcome 3). Table S4. Sensitivity analysis for individual enrolment (outcome 1). Table S5. Sensitivity analysis for household enrolment (outcome 2). Table S6. Results of the multivariate logistic regression for individual enrolment (outcome 1). Table S7. Results of the multivariate logistic regression for household enrolment (outcome 2). Table S8. Results of the multivariate logistic regression for complete household enrolment (outcome 3). [file 12913_2023_10473_MOESM1_ESM.pdf]

Table S1: Results of the bivariate analysis for individual enrolment (outcome 1)

	Pooled					Bihar					Uttarakhand				
	enrolled		not enrolled		Chi 2	enrolled		not enrolled		Chi 2	enrolled		not enrolled		Chi 2
	n	%	n	%		n	%	n	%		n	%	n	%	
sex ind															
...male	9019	48.0	9766	52.0	0.192	1109	43.0	1468	57.0	0.371	426	17.7	1976	82.3	0.152
...female	8619	48.7	9081	51.3		948	41.8	1322	58.2		424	19.4	1764	80.6	
age ind															
...0-14	4129	40.4	6094	59.6	0.000	797	39.6	1217	60.4	0.003	199	15.5	1085	84.5	0.004
...15-49	10019	50.3	9905	49.7		931	44.1	1178	55.9		516	19.5	2127	80.5	
...50+	3490	55.1	2848	44.9		329	45.4	395	54.6		135	20.4	528	79.6	
relationship to hoh															
... hoh	4243	55.8	3359	44.2	0.000	448	49.8	452	50.2	0.000	191	21.2	709	78.8	0.001
... spouse	3391	54.2	2871	45.9		409	51.7	382	48.3		165	21.4	608	78.7	
... child	7697	46.3	8912	53.7		1072	42.0	1482	58.0		408	17.7	1900	82.3	
... others	2307	38.4	3705	61.6		128	21.3	474	78.7		86	14.1	523	85.9	
caste															
...No/ other/ general caste	3494	49.1	3625	50.9	0.000	432	43.8	554	56.2	0.001	86	15.3	477	84.7	0.001
...scheduled caste/ tribe	8468	53.4	7398	46.6		486	46.6	556	53.4		373	21.1	1396	78.9	
...other backward class	5603	41.9	7777	58.1		1139	40.4	1680	59.6		391	17.3	1867	82.7	
religion															
...hindu	10702	43.8	13749	56.2	0.000	1728	42.6	2326	57.4	0.100	582	20.3	2290	79.7	0.000
...muslim	2757	42.1	3795	57.9		329	41.8	458	58.2		228	14.3	1364	85.7	
...christian/ other	4179	76.2	1303	23.8		0	0.0	6	100.0		40	31.8	86	68.3	
education ind															
...no education	4748	39.3	7327	60.7	0.000	996	39.8	1509	60.2	0.000	292	17.5	1381	82.6	0.118
...up to 7 years	6644	51.6	6224	48.4		750	45.8	886	54.2		254	18.0	1157	82.0	
...8 years and above	6246	54.1	5296	45.9		311	44.1	395	56.0		304	20.2	1202	79.8	
occupation ind															
...farming	2628	54.9	2158	45.1	0.000	155	46.7	177	53.3	0.000	20	13.5	128	86.5	0.000
...labourer/ daily wage worker	2881	53.2	2536	46.8		299	44.1	379	55.9		194	20.9	736	79.1	
...other occup	1167	51.7	1089	48.3		116	42.0	160	58.0		63	17.5	297	82.5	
...student	4742	46.0	5561	54.0		780	46.2	910	53.9		265	18.3	1184	81.7	
...housewife	3791	46.7	4323	53.3		466	45.0	569	55.0		241	20.1	961	80.0	
...not employed	1709	55.5	1370	44.5		103	38.0	168	62.0		47	19.4	195	80.6	
...not yet in school	720	28.5	1810	71.5		138	24.4	427	75.6		20	7.7	239	92.3	
below poverty line															
...yes	11710	49.1	12122	50.9	0.000	1757	43.8	2258	56.2	0.000	586	19.7	2387	80.3	0.005
...no	5928	46.9	6725	53.2		300	36.1	532	63.9		264	16.3	1353	83.7	
wealth index															
...lowest	3219	49.5	3287	50.5	0.000	363	38.2	588	61.8	0.001	179	27.0	484	73.0	0.000
...second	3567	48.6	3775	51.4		411	45.5	493	54.5		162	16.1	845	83.9	
...third	3695	49.4	3780	50.6		447	43.8	573	56.2		142	14.8	815	85.2	
...fourth	3548	48.3	3802	51.7		375	39.1	583	60.9		170	17.3	812	82.7	
...highest	3609	46.2	4203	53.8		461	45.5	553	54.5		197	20.1	784	79.9	
state (only for pooled sample)															
...Bihar	2057	42.4	2790	57.6	0.000	-					-				
...Uttarakhand	850	18.5	3740	81.5		-					-				
...Uttar Pradesh	1087	20.5	4219	79.5		-					-				
...West Bengal	1347	37.9	2212	62.2		-					-				
...Gujarat	2107	40.9	3047	59.1		-					-				
...Kerala	3422	79.8	865	20.2		-					-				
...Mizoram	3541	79.7	900	20.3		-					-				
...Tripura	3227	75.0	1074	25.0		-					-				
	mean	SD	mean	SD	t-test	mean	SD	mean	SD	t-test	mean	SD	mean	SD	t-test
household size	5.07	1.64	6.04	2.32	0.000	5.90	1.83	6.36	2.14	0.000	5.59	1.91	5.88	2.07	0.0000

	Uttar Pradesh					West Bengal					Gujarat				
	enrolled		not enrolled		Chi 2	enrolled		not enrolled		Chi 2	enrolled		not enrolled		Chi 2
	n	%	n	%		n	%	n	%		n	%	n	%	
sex ind															
...male	584	20.8	2219	79.2	0.506	708	38.5	1130	61.5	0.393	1115	41.6	1568	58.4	0.303
...female	503	20.1	2000	79.9		639	37.1	1082	62.9		992	40.2	1479	59.9	
age ind															
...0-14	320	17.7	1485	82.3	0.002	271	33.1	547	66.9	0.003	397	30.9	886	69.1	0.000
...15-49	592	21.9	2117	78.2		836	39.9	1258	60.1		1329	44.2	1677	55.8	
...50+	175	22.1	617	77.9		240	37.1	407	62.9		381	44.1	484	56.0	
relationship to hoh															
... hoh	230	25.5	671	74.5	0.000	357	39.8	540	60.2	0.023	478	47.4	530	52.6	0.000
... spouse	196	24.9	590	75.1		325	40.0	488	60.0		411	48.8	432	51.3	
... child	532	20.6	2045	79.4		509	37.3	857	62.7		780	38.3	1255	61.7	
... others	129	12.4	913	87.6		156	32.3	327	67.7		438	34.5	830	65.5	
caste															
...No/ other/ general caste	74	28.9	182	71.1	0.000	696	30.4	1592	69.6	0.000	105	55.3	85	44.7	0.000
...scheduled caste/ tribe	638	19.1	2699	80.9		527	56.4	408	43.6		579	42.9	770	57.1	
...other backward class	373	21.8	1338	78.2		120	37.2	203	62.9		1423	39.6	2168	60.4	
religion															
...hindu	1017	20.5	3946	79.5	0.043	792	42.6	1069	57.4		2075	41.1	2969	58.9	0.001
...muslim	70	21.9	250	78.1		546	32.3	1143	67.7		14	20.0	56	80.0	
...christian/ other	0	0.0	23	100.0		9	100.0	0	0.0		18	45.0	22	55.0	
education ind															
...no education	449	20.0	1793	80.0	0.538	554	41.8	773	58.3	0.000	760	38.1	1235	61.9	0.000
...up to 7 years	329	20.2	1297	79.8		511	38.7	808	61.3		747	40.9	1078	59.1	
...8 years and above	309	21.5	1129	78.5		282	30.9	631	69.1		600	45.0	734	55.0	
occupation ind															
...farming	197	29.1	480	70.9	0.000	316	42.5	428	57.5	0.001	571	46.5	656	53.5	0.000
...labourer/ daily wage worker	102	17.7	476	82.4		192	36.9	328	63.1		185	45.1	225	54.9	
...other occup	34	15.3	189	84.8		73	44.5	91	55.5		78	43.6	101	56.4	
...student	371	20.6	1428	79.4		321	35.4	585	64.6		472	39.0	739	61.0	
...housewife	295	21.8	1057	78.2		319	37.8	524	62.2		542	42.9	723	57.2	
...not employed	48	19.0	205	81.0		80	38.5	128	61.5		200	38.4	321	61.6	
...not yet in school	40	9.4	385	90.6		46	26.4	128	73.6		59	17.3	282	82.7	
below poverty line															
...yes	686	17.6	3209	82.4	0.000	1068	69.1	478	30.9	0.000	1224	36.1	2167	63.9	0.000
...no	401	28.4	1010	71.6		279	13.9	1734	86.1		883	50.1	880	49.9	
wealth index															
...lowest	235	24.5	725	75.5	0.001	285	40.7	415	59.3	0.000	303	36.2	534	63.8	0.000
...second	185	18.1	835	81.9		334	44.8	412	55.2		320	32.0	681	68.0	
...third	182	17.5	858	82.5		321	44.1	407	55.9		484	43.9	618	56.1	
...fourth	234	21.3	865	78.7		216	33.5	429	66.5		475	46.5	547	53.5	
...highest	251	21.2	936	78.9		191	25.8	549	74.2		525	44.0	667	56.0	
state (only for pooled sample)															
...Bihar	-					-					-				
...Uttarakhand	-					-					-				
...Uttar Pradesh	-					-					-				
...West Bengal	-					-					-				
...Gujarat	-					-					-				
...Kerala	-					-					-				
...Mizoram	-					-					-				
...Tripura	-					-					-				
	mean	SD	mean	SD	t-test	mean	SD	mean	SD	t-test	mean	SD	mean	SD	t-test
household size	6.13	2.06	7.16	2.87	0.000	4.38	1.25	4.55	1.71	0.002	5.62	1.72	6.01	1.96	0.000

	Kerala					Mizoram					Tripura				
	enrolled		not enrolled		Chi 2	enrolled		not enrolled		Chi 2	enrolled		not enrolled		Chi 2
	n	%	n	%		n	%	n	%		n	%	n	%	
sex ind															
...male	1626	80.0	407	20.0	0.807	1750	79.2	459	20.8	0.398	1701	75.9	539	24.1	0.151
...female	1796	79.7	458	20.3		1791	80.2	441	19.8		1526	74.0	535	26.0	
age ind															
...0-14	523	69.0	235	31.0	0.000	940	74.5	322	25.5	0.000	682	68.3	317	31.7	0.000
...15-49	1914	79.9	481	20.1		1984	81.6	449	18.5		1917	75.6	618	24.4	
...50+	985	86.9	149	13.1		617	82.7	129	17.3		628	81.9	139	18.1	
relationship to hoh															
... hoh	860	85.6	145	14.4	0.000	843	84.4	156	15.6	0.000	836	84.3	156	15.7	0.000
... spouse	622	84.4	115	15.6		587	83.6	115	16.4		676	82.7	141	17.3	
... child	1296	80.2	320	19.8		1686	79.0	449	21.0		1414	70.1	604	29.9	
... others	644	69.3	285	30.7		425	70.3	180	29.8		301	63.5	173	36.5	
caste															
...No/ other/ general caste	1012	75.4	330	24.6	0.000	20	100.0	0	0.0	0.077	1069	72.5	405	27.5	0.011
...scheduled caste/ tribe	867	82.5	184	17.5		3516	79.7	895	20.3		1482	75.2	490	24.9	
...other backward class	1539	81.5	349	18.5		5	83.3	1	16.7		613	78.2	171	21.8	
religion															
...hindu	1977	80.9	466	19.1	0.000	30	75.0	10	25.0	0.353	2501	78.8	673	21.2	0.000
...muslim	1043	81.0	244	19.0		6	100.0	0	0.0		521	65.0	280	35.0	
...christian/ other	402	72.2	155	27.8		3505	79.8	890	20.3		205	62.9	121	37.1	
education ind															
...no education	334	67.8	159	32.3	0.000	570	72.5	216	27.5	0.000	793	75.2	261	24.8	0.581
...up to 7 years	1108	84.3	207	15.7		1667	81.4	381	18.6		1278	75.7	410	24.3	
...8 years and above	1980	79.9	499	20.1		1304	81.1	303	18.9		1156	74.2	403	25.9	
occupation ind															
...farming	179	78.9	48	21.2	0.000	865	83.1	176	16.9	0.000	325	83.8	63	16.2	0.000
...labourer/ daily wage worker	760	83.0	156	17.0		362	84.6	66	15.4		787	82.2	170	17.8	
...other occup	352	80.7	84	19.3		255	75.9	81	24.1		196	69.5	86	30.5	
...student	719	81.0	169	19.0		915	79.2	240	20.8		899	74.5	308	25.5	
...housewife	810	81.3	186	18.7		491	82.0	108	18.0		627	76.2	196	23.8	
...not employed	525	82.7	110	17.3		394	79.8	100	20.2		312	68.6	143	31.4	
...not yet in school	77	40.7	112	59.3		259	66.8	129	33.3		81	42.9	108	57.1	
below poverty line															
...yes	2535	79.6	648	20.4	0.616	1390	85.3	240	14.7	0.000	2464	77.0	735	23.0	0.000
...no	887	80.3	217	19.7		2151	76.5	660	23.5		763	69.2	339	30.8	
wealth index															
...lowest	602	81.4	138	18.7	0.092	719	75.0	240	25.0	0.000	533	76.6	163	23.4	0.005
...second	676	79.5	174	20.5		816	85.6	137	14.4		663	77.0	198	23.0	
...third	701	82.2	152	17.8		760	84.4	141	15.7		658	75.3	216	24.7	
...fourth	736	79.2	193	20.8		682	79.8	173	20.2		660	76.7	200	23.3	
...highest	707	77.3	208	22.7		564	73.0	209	27.0		713	70.6	297	29.4	
state (only for pooled sample)															
...Bihar	-					-					-				
...Uttarakhand	-					-					-				
...Uttar Pradesh	-					-					-				
...West Bengal	-					-					-				
...Gujarat	-					-					-				
...Kerala	-					-					-				
...Mizoram	-					-					-				
...Tripura	-					-					-				
	mean	SD	mean	SD	t-test	mean	SD	mean	SD	t-test	mean	SD	mean	SD	t-test
household size	4.66	1.34	5.14	1.57	0.000	4.72	1.22	5.61	1.57	0.000	4.81	1.58	5.56	1.86	0.000

Note: Sex was the key variable of interest. The remaining covariates were not discussed in the paper.
Abbreviation: ind= individual, hoh = head of household, SD = standard deviation.

Table S2: Results of the bivariate analysis for household enrolment (outcome 2)

	Pooled					Bihar					Uttarakhand				
	enrolled		not enrolled		Chi 2	enrolled		not enrolled		Chi 2	enrolled		not enrolled		Chi 2
	n	%	n	%		n	%	n	%		n	%	n	%	
sex hoh															
...male	3829	56.6	2937	43.4	0.000	448	54.2	378	45.8	0.461	185	22.3	644	77.7	0.096
...female	610	72.4	233	27.6		44	58.7	31	41.3		22	31.0	49	69.0	
age hoh															
...0-14	0	0.0	0	0.0	0.001	0	0.0	0	0.0	0.049	0	0.0	0	0.0	0.906
...15-49	2526	56.8	1925	43.3		292	57.5	216	42.5		127	23.1	422	76.9	
...50+	1913	60.6	1245	39.4		200	50.9	193	49.1		80	22.8	271	77.2	
caste															
...No/ other/ general caste	929	56.3	722	43.7	0.000	104	56.8	79	43.2	0.356	21	18.1	95	81.9	0.272
...scheduled caste/ tribe	2102	63.3	1218	36.7		114	57.9	83	42.1		89	25.2	264	74.8	
...other backward class	1391	53.3	1221	46.8		274	52.6	247	47.4		97	22.5	334	77.5	
religion															
...hindu	2713	53.8	2332	46.2	0.000	412	53.9	352	46.1	0.313	144	24.0	457	76.0	0.214
...muslim	696	52.3	634	47.7		80	58.8	56	41.2		54	19.9	217	80.1	
...christian/ other	1030	83.5	204	16.5		0	0.0	1	100.0		9	32.1	19	67.9	
education hoh															
...no education	1346	48.7	1416	51.3	0.000	305	53.6	264	46.4	0.730	93	21.3	344	78.7	0.492
...up to 7 years	1671	66.8	831	33.2		86	56.2	67	43.8		48	24.7	146	75.3	
...8 years and above	1422	60.6	923	39.4		101	56.4	78	43.6		66	24.5	203	75.5	
occupation hoh															
...farming	1671	56.7	1278	43.3	0.000	118	47.4	131	52.6	0.095	19	17.0	93	83.0	0.549
...labourer/ daily wage worker	1618	57.3	1208	42.8		232	56.3	180	43.7		132	24.5	407	75.5	
...other occupation	637	59.2	439	40.8		86	59.3	59	40.7		35	21.5	128	78.5	
...student	4	50.0	4	50.0		0	0.0	0	0.0		0	0.0	1	100.0	
...housewife	151	61.9	93	38.1		22	56.4	17	43.6		7	21.2	26	78.8	
...not employed	358	70.8	148	29.3		34	60.7	22	39.3		14	26.9	38	73.1	
...not yet in school	0	0.0	0	0.0		0	0.0	0	0.0		0	0.0	0	0.0	
below poverty line															
...yes	2973	60.6	1935	39.4	0.000	417	56.0	328	44.0	0.072	143	23.9	456	76.1	0.380
...no	1466	54.3	1235	45.7		75	48.1	81	51.9		64	21.3	237	78.7	
wealth index															
...lowest	860	57.5	637	42.6	0.838	88	47.3	98	52.7	0.180	43	30.3	99	69.7	0.062
...second	905	58.1	652	41.9		101	57.1	76	42.9		39	20.6	150	79.4	
...third	913	59.4	624	40.6		103	56.0	81	44.0		35	18.7	152	81.3	
...fourth	879	58.8	617	41.2		96	53.6	83	46.4		39	20.4	152	79.6	
...highest	882	58.0	640	42.1		104	59.4	71	40.6		51	26.7	140	73.3	
state (only for pooled sample)															
...Bihar	492	54.6	409	45.4	0.000	-					-				
...Uttarakhand	207	23.0	693	77.0		-					-				
...Uttar Pradesh	256	28.4	646	71.6		-					-				
...West Bengal	372	41.4	527	58.6		-					-				
...Gujarat	503	49.9	505	50.1		-					-				
...Kerala	891	88.7	114	11.3		-					-				
...Mizoram	854	85.5	145	14.5		-					-				
...Tripura	864	86.8	131	13.2		-					-				
	mean	SD	mean	SD	t-test	mean	SD	mean	SD	t-test	mean	SD	mean	SD	t-test
household size	4.70	1.76	4.98	2.08	0.000	5.49	2.03	5.27	2.06	0.106	5.19	2.00	5.08	1.90	0.469

	Uttar Pradesh					West Bengal					Gujarat				
	enrolled		not enrolled		Chi 2	enrolled		not enrolled		Chi 2	enrolled		not enrolled		Chi 2
	n	%	n	%		n	%	n	%		n	%	n	%	
sex hoh															
...male	241	28.5	606	71.6	0.851	341	40.5	502	59.6	0.028	476	50.2	473	49.8	0.512
...female	15	27.3	40	72.7		31	55.4	25	44.6		27	45.8	32	54.2	
age hoh															
...0-14	0	0.0	0	0.0	0.076	0	0.0	0	0.0	0.145	0	0.0	0	0.0	0.742
...15-49	167	30.5	380	69.5		245	43.2	322	56.8		381	50.2	378	49.8	
...50+	89	25.1	266	74.9		127	38.3	205	61.8		122	49.0	127	51.0	
caste															
...No/ other/ general caste	18	37.5	30	62.5	0.038	198	33.7	389	66.3	0.000	27	64.3	15	35.7	0.144
...scheduled caste/ tribe	146	25.6	425	74.4		142	60.4	93	39.6		142	51.1	136	48.9	
...other backward class	92	32.5	191	67.5		31	41.9	43	58.1		334	48.9	349	51.1	
religion															
...hindu	240	28.3	607	71.7	0.513	220	45.5	264	54.6	0.005	493	50.0	494	50.1	0.312
...muslim	16	30.8	36	69.2		150	36.3	263	63.7		4	33.3	8	66.7	
...christian/ other	0	0.0	3	100.0		2	100.0	0	0.0		6	66.7	3	33.3	
education hoh															
...no education	126	27.5	333	72.6	0.036	204	45.4	245	54.6	0.016	151	46.9	171	53.1	0.275
...up to 7 years	35	22.2	123	77.9		104	40.3	154	59.7		174	49.6	177	50.4	
...8 years and above	95	33.3	190	66.7		64	33.3	128	66.7		178	53.1	157	46.9	
occupation hoh															
...farming	142	32.1	300	67.9	0.010	220	42.5	298	57.5	0.525	333	51.1	319	48.9	0.695
...labourer/ daily wage worker	62	21.5	227	78.6		97	39.1	151	60.9		118	48.8	124	51.2	
...other occupation	32	34.0	62	66.0		40	41.7	56	58.3		40	44.0	51	56.0	
...student	0	0.0	2	100.0		1	100.0	0	0.0		1	100.0	0	0.0	
...housewife	14	32.6	29	67.4		7	53.9	6	46.2		3	60.0	2	40.0	
...not employed	6	18.8	26	81.3		7	30.4	16	69.6		8	47.1	9	52.9	
...not yet in school	0	0.0	0	0.0		0	0.0	0	0.0		0	0.0	0	0.0	
below poverty line															
...yes	164	25.4	483	74.7	0.001	298	76.2	93	23.8	0.000	301	44.7	372	55.3	0.000
...no	92	36.1	163	63.9		74	14.6	434	85.4		202	60.3	133	39.7	
wealth index															
...lowest	52	29.1	127	71.0	0.083	88	45.1	107	54.9	0.000	80	40.6	117	59.4	0.001
...second	44	23.5	143	76.5		93	48.7	98	51.3		81	42.4	110	57.6	
...third	44	23.8	141	76.2		83	46.6	95	53.4		112	54.4	94	45.6	
...fourth	56	31.6	121	68.4		57	35.4	104	64.6		111	57.8	81	42.2	
...highest	60	34.5	114	65.5		51	29.3	123	70.7		119	53.6	103	46.4	
state (only for pooled sample)															
...Bihar	-					-					-				
...Uttarakhand	-					-					-				
...Uttar Pradesh	-					-					-				
...West Bengal	-					-					-				
...Gujarat	-					-					-				
...Kerala	-					-					-				
...Mizoram	-					-					-				
...Tripura	-					-					-				
	mean	SD	mean	SD	t-test	mean	SD	mean	SD	t-test	mean	SD	mean	SD	t-test
household size	5.93	2.47	5.89	2.50	0.800	4.08	1.32	3.90	1.51	0.058	5.27	1.87	4.98	1.98	0.019

	Kerala					Mizoram					Tripura				
	enrolled		not enrolled		Chi 2	enrolled		not enrolled		Chi 2	enrolled		not enrolled		Chi 2
	n	%	n	%		n	%	n	%		n	%	n	%	
sex hoh															
...male	689	88.0	94	12.0	0.214	681	84.5	125	15.5	0.068	768	87.0	115	13.0	0.710
...female	202	91.0	20	9.0		173	89.6	20	10.4		96	85.7	16	14.3	
age hoh															
...0-14	0	0.0	0	0.0	0.011	0	0.0	0	0.0	0.113	0	0.0	0	0.0	0.531
...15-49	351	85.6	59	14.4		467	87.1	69	12.9		496	86.3	79	13.7	
...50+	540	90.8	55	9.2		387	83.6	76	16.4		368	87.6	52	12.4	
caste															
...No/ other/ general caste	275	82.6	58	17.4	0.000	5	100.0	0	0.0	0.601	283	83.5	56	16.5	0.081
...scheduled caste/ tribe	219	90.5	23	9.5		848	85.5	144	14.5		400	88.9	50	11.1	
...other backward class	395	92.3	33	7.7		1	100.0	0	0.0		167	87.4	24	12.6	
religion															
...hindu	523	87.9	72	12.1	0.000	7	87.5	1	12.5	0.906	674	88.8	85	11.2	0.000
...muslim	260	94.2	16	5.8		1	100.0	0	0.0		131	77.5	38	22.5	
...christian/ other	108	80.6	26	19.4		846	85.5	144	14.6		59	88.1	8	11.9	
education hoh															
...no education	100	94.3	6	5.7	0.000	83	84.7	15	15.3	0.604	284	88.2	38	11.8	0.674
...up to 7 years	394	92.5	32	7.5		497	86.4	78	13.6		333	86.1	54	14.0	
...8 years and above	397	83.9	76	16.1		274	84.1	52	16.0		247	86.4	39	13.6	
occupation hoh															
...farming	130	83.3	26	16.7	0.046	457	85.4	78	14.6	0.283	252	88.4	33	11.6	0.069
...labourer/ daily wage worker	379	89.8	43	10.2		163	90.1	18	9.9		435	88.2	58	11.8	
...other occupation	180	87.0	27	13.0		120	80.5	29	19.5		104	79.4	27	20.6	
...student	1	50.0	1	50.0		1	100.0	0	0.0		0	0.0	0	0.0	
...housewife	67	91.8	6	8.2		20	83.3	4	16.7		11	78.6	3	21.4	
...not employed	134	92.4	11	7.6		93	85.3	16	14.7		62	86.1	10	13.9	
...not yet in school	0	0.0	0	0.0		0	0.0	0	0.0		0	0.0	0	0.0	
below poverty line															
...yes	649	88.1	88	11.9	0.322	342	90.7	35	9.3	0.000	659	89.2	80	10.8	0.000
...no	242	90.3	26	9.7		512	82.3	110	17.7		205	80.1	51	19.9	
wealth index															
...lowest	170	86.7	26	13.3	0.336	182	81.6	41	18.4	0.009	157	87.7	22	12.3	0.982
...second	179	87.8	25	12.3		194	90.2	21	9.8		174	85.7	29	14.3	
...third	179	92.8	14	7.3		185	89.4	22	10.6		172	87.3	25	12.7	
...fourth	186	89.0	23	11.0		159	85.5	27	14.5		175	87.1	26	12.9	
...highest	177	87.2	26	12.8		134	79.8	34	20.2		186	86.5	29	13.5	
state (only for pooled sample)															
...Bihar	-					-					-				
...Uttarakhand	-					-					-				
...Uttar Pradesh	-					-					-				
...West Bengal	-					-					-				
...Gujarat	-					-					-				
...Kerala	-					-					-				
...Mizoram	-					-					-				
...Tripura	-					-					-				
	mean	SD	mean	SD	t-test	mean	SD	mean	SD	t-test	mean	SD	mean	SD	t-test
household size	4.28	1.47	4.16	1.28	0.395	4.40	1.34	4.88	1.62	0.000	4.44	1.60	4.32	1.78	0.443

Note: Sex was the key variable of interest. The remaining covariates were not discussed in the paper.
Abbreviation: hoh = head of household, SD = standard deviation.

Table S3: Results of the bivariate analysis for complete household enrolment (outcome 3)

	Pooled					Bihar					Uttarakhand				
	enrolled		not enrolled		Chi 2	enrolled		not enrolled		Chi 2	enrolled		not enrolled		Chi 2
	n	%	n	%		n	%	n	%		n	%	n	%	
sex hoh															
...male	3118	81.4	711	18.6	0.075	337	75.2	111	24.8	0.974	154	83.2	31	16.8	0.224
...female	515	84.4	95	15.6		33	75.0	11	25.0		16	72.7	6	27.3	
age hoh															
...0-14	0	0.0	0	0.0	0.959	0	0.0	0	0.0	0.931	0	0.0	0	0.0	0.314
...15-49	2068	81.9	458	18.1		220	75.3	72	24.7		107	84.3	20	15.8	
...50+	1565	81.8	348	18.2		150	75.0	50	25.0		63	78.8	17	21.3	
caste															
...No/ other/ general caste	744	79.9	187	20.1	0.000	81	77.9	23	22.1	0.327	18	85.7	3	14.3	0.619
...scheduled caste/ tribe	1793	85.4	307	14.6		90	79.0	24	21.1		75	84.3	14	15.7	
...other backward class	1083	77.9	308	22.1		199	72.6	75	27.4		77	79.4	20	20.6	
religion															
...hindu	2173	80.1	540	19.9	0.000	314	76.2	98	23.8	0.239	123	85.4	21	14.6	0.084
...muslim	533	76.6	163	23.4		56	70.0	24	30.0		39	72.2	15	27.8	
...christian/ other	927	90.0	103	10.0		0	0.0	0	0.0		8	88.9	1	11.1	
education hoh															
...no education	1068	79.4	278	20.7	0.017	224	73.4	81	26.6	0.290	78	83.9	15	16.1	0.580
...up to 7 years	1389	83.1	282	16.9		64	74.4	22	25.6		37	77.1	11	22.9	
...8 years and above	1176	82.7	246	17.3		82	81.2	19	18.8		55	83.3	11	16.7	
occupation hoh															
...farming	1378	82.5	293	17.5	0.732	80	67.8	38	32.2	0.134	16	84.2	3	15.8	0.726
...labourer/ daily wage worker	1318	81.5	300	18.5		180	77.6	52	22.4		111	84.1	21	15.9	
...other occup	523	82.1	114	17.9		70	81.4	16	18.6		26	74.3	9	25.7	
...student	3	75.0	1	25.0		0	0.0	0	0.0		0	0.0	0	0.0	
...housewife	127	84.1	24	15.9		17	77.3	5	22.7		6	85.7	1	14.3	
...not employed	284	79.3	74	20.7		23	67.7	11	32.4		11	78.6	3	21.4	
...not yet in school	0	0.0	0	0.0		0	0.0	0	0.0		0	0.0	0	0.0	
below poverty line															
...yes	2399	80.7	574	19.3	0.005	310	74.3	107	25.7	0.296	119	83.2	24	16.8	0.540
...no	1234	84.2	232	15.8		60	80.0	15	20.0		51	79.7	13	20.3	
wealth index															
...lowest	737	85.7	123	14.3	0.000	68	77.3	20	22.7	0.217	40	93.0	3	7.0	0.080
...second	745	82.3	160	17.7		73	72.3	28	27.7		30	76.9	9	23.1	
...third	768	84.1	145	15.9		85	82.5	18	17.5		27	77.1	8	22.9	
...fourth	701	79.8	178	20.3		66	68.8	30	31.3		35	89.7	4	10.3	
...highest	682	77.3	200	22.7		78	75.0	26	25.0		38	74.5	13	25.5	
state (only for pooled sample)															
...Bihar	370	75.2	122	24.8	0.000	-					-				
...Uttarakhand	170	82.1	37	17.9		-					-				
...Uttar Pradesh	195	76.2	61	23.8		-					-				
...West Bengal	293	78.8	79	21.2		-					-				
...Gujarat	410	81.5	93	18.5		-					-				
...Kerala	742	83.3	149	16.7		-					-				
...Mizoram	801	93.8	53	6.2		-					-				
...Tripura	652	75.5	212	24.5		-					-				
	mean	SD	mean	SD	t-test	mean	SD	mean	SD	t-test	mean	SD	mean	SD	t-test
household size	4.59	1.74	5.20	1.76	0.000	5.49	2.09	5.48	1.84	0.969	5.11	2.08	5.57	1.56	0.205

	Uttar Pradesh					West Bengal					Gujarat				
	enrolled		not enrolled		Chi 2	enrolled		not enrolled		Chi 2	enrolled		not enrolled		Chi 2
	n	%	n	%		n	%	n	%		n	%	n	%	
sex hoh															
...male	183	75.9	58	24.1	0.720	269	78.9	72	21.1	0.848	388	81.5	88	18.5	0.997
...female	12	80.0	3	20.0		24	77.4	7	22.6		22	81.5	5	18.5	
age hoh															
...0-14	0	0.0	0	0.0	0.074	0	0.0	0	0.0	0.795	0	0.0	0	0.0	0.513
...15-49	133	79.6	34	20.4		192	78.4	53	21.6		313	82.2	68	17.9	
...50+	62	69.7	27	30.3		101	79.5	26	20.5		97	79.5	25	20.5	
caste															
...No/ other/ general caste	15	83.3	3	16.7	0.273	152	76.8	46	23.2	0.621	21	77.8	6	22.2	0.850
...scheduled caste/ tribe	115	78.8	31	21.2		115	81.0	27	19.0		117	82.4	25	17.6	
...other backward class	65	70.7	27	29.4		25	80.7	6	19.4		272	81.4	62	18.6	
religion															
...hindu	183	76.3	57	23.8	0.910	174	79.1	46	20.9	0.739	406	82.4	87	17.7	0.002
...muslim	12	75.0	4	25.0		117	78.0	33	22.0		1	25.0	3	75.0	
...christian/ other	0	0.0	0	0.0		2	100.0	0	0.0		3	50.0	3	50.0	
education hoh															
...no education	99	78.6	27	21.4	0.409	164	80.4	40	19.6	0.539	121	80.1	30	19.9	0.766
...up to 7 years	28	80.0	7	20.0		78	75.0	26	25.0		141	81.0	33	19.0	
...8 years and above	68	71.6	27	28.4		51	79.7	13	20.3		148	83.2	30	16.9	
occupation hoh															
...farming	114	80.3	28	19.7	0.030	173	78.6	47	21.4	0.778	267	80.2	66	19.8	0.571
...labourer/ daily wage worker	49	79.0	13	21.0		77	79.4	20	20.6		102	86.4	16	13.6	
...other occup	21	65.6	11	34.4		32	80.0	8	20.0		31	77.5	9	22.5	
...student	0	0.0	0	0.0		1	100.0	0	0.0		1	100.0	0	0.0	
...housewife	9	64.3	5	35.7		4	57.1	3	42.9		3	100.0	0	0.0	
...not employed	2	33.3	4	66.7		6	85.7	1	14.3		6	75.0	2	25.0	
...not yet in school	0	0.0	0	0.0		0	0.0	0	0.0		0	0.0	0	0.0	
below poverty line															
...yes	124	75.6	40	24.4	0.778	237	79.5	61	20.5	0.468	238	79.1	63	20.9	0.085
...no	71	77.2	21	22.8		56	75.7	18	24.3		172	85.2	30	14.9	
wealth index															
...lowest	46	88.5	6	11.5	0.049	68	77.3	20	22.7	0.500	72	90.0	8	10.0	0.286
...second	32	72.7	12	27.3		70	75.3	23	24.7		63	77.8	18	22.2	
...third	36	81.8	8	18.2		70	84.3	13	15.7		89	79.5	23	20.5	
...fourth	42	75.0	14	25.0		47	82.5	10	17.5		89	80.2	22	19.8	
...highest	39	65.0	21	35.0		38	74.5	13	25.5		97	81.5	22	18.5	
state (only for pooled sample)															
...Bihar	-					-					-				
...Uttarakhand	-					-					-				
...Uttar Pradesh	-					-					-				
...West Bengal	-					-					-				
...Gujarat	-					-					-				
...Kerala	-					-					-				
...Mizoram	-					-					-				
...Tripura	-					-					-				
	mean	SD	mean	SD	t-test	mean	SD	mean	SD	t-test	mean	SD	mean	SD	t-test
household size	5.56	2.03	7.13	3.27	0.000	3.94	1.31	4.62	1.21	0.000	5.20	1.94	5.57	1.45	0.084

	Kerala					Mizoram					Tripura				
	enrolled		not enrolled		Chi 2	enrolled		not enrolled		Chi 2	enrolled		not enrolled		Chi 2
	n	%	n	%		n	%	n	%		n	%	n	%	
sex hoh															
...male	573	83.2	116	16.8	0.867	638	93.7	43	6.3	0.795	576	75.0	192	25.0	0.371
...female	169	83.7	33	16.3		163	94.2	10	5.8		76	79.2	20	20.8	
age hoh															
...0-14	0	0.0	0	0.0	0.219	0	0.0	0	0.0	0.390	0	0.0	0	0.0	0.397
...15-49	299	85.2	52	14.8		435	93.2	32	6.9		369	74.4	127	25.6	
...50+	443	82.0	97	18.0		366	94.6	21	5.4		283	76.9	85	23.1	
caste															
...No/ other/ general caste	232	84.4	43	15.6	0.088	4	80.0	1	20	0.426	221	78.1	62	21.9	0.390
...scheduled caste/ tribe	191	87.2	28	12.8		796	93.9	52	6.1		294	73.5	106	26.5	
...other backward class	318	80.5	77	19.5		1	100.0	0	0.0		126	75.5	41	24.6	
religion															
...hindu	447	85.5	76	14.5	0.100	6	85.7	1	14.3	0.652	520	77.2	154	22.9	0.009
...muslim	210	80.8	50	19.2		1	100.0	0	0.0		97	74.1	34	26.0	
...christian/ other	85	78.7	23	21.3		794	93.9	52	6.2		35	59.3	24	40.7	
education hoh															
...no education	83	83.0	17	17.0	0.384	82	98.8	1	1.2	0.076	217	76.4	67	23.6	0.738
...up to 7 years	321	81.5	73	18.5		467	94.0	30	6.0		253	76.0	80	24.0	
...8 years and above	338	85.1	59	14.9		252	92.0	22	8.0		182	73.7	65	26.3	
occupation hoh															
...farming	105	80.8	25	19.2	0.403	430	94.1	27	5.9	0.005	193	76.6	59	23.4	0.364
...labourer/ daily wage worker	312	82.3	67	17.7		152	93.3	11	6.8		335	77.0	100	23.0	
...other occup	156	86.7	24	13.3		112	93.3	8	6.7		75	72.1	29	27.9	
...student	1	100.0	0	0.0		0	0.0	1	100.0		0	0.0	0	0.0	
...housewife	60	89.6	7	10.5		20	100.0	0	0.0		8	72.7	3	27.3	
...not employed	108	80.6	26	19.4		87	93.6	6	6.5		41	66.1	21	33.9	
...not yet in school	0	0.0	0	0.0		0	0.0	0	0.0		0	0.0	0	0.0	
below poverty line															
...yes	550	84.8	99	15.3	0.054	321	93.9	21	6.1	0.948	500	75.9	159	24.1	0.616
...no	192	79.3	50	20.7		480	93.8	32	6.3		152	74.2	53	25.9	
wealth index															
...lowest	156	91.8	14	8.2	0.002	171	94.0	11	6.0	0.844	116	73.9	41	26.1	0.064
...second	149	83.2	30	16.8		185	95.4	9	4.6		143	82.2	31	17.8	
...third	154	86.0	25	14.0		173	93.5	12	6.5		134	77.9	38	22.1	
...fourth	145	78.0	41	22.0		147	92.5	12	7.6		130	74.3	45	25.7	
...highest	138	78.0	39	22.0		125	93.3	9	6.7		129	69.4	57	30.7	
state (only for pooled sample)															
...Bihar	-					-					-				
...Uttarakhand	-					-					-				
...Uttar Pradesh	-					-					-				
...West Bengal	-					-					-				
...Gujarat	-					-					-				
...Kerala	-					-					-				
...Mizoram	-					-					-				
...Tripura	-					-					-				
	mean	SD	mean	SD	t-test	mean	SD	mean	SD	t-test	mean	SD	mean	SD	t-test
household size	4.18	1.48	4.78	1.30	0.000	4.38	1.35	4.74	1.11	0.063	4.29	1.64	4.89	1.38	0.000

Note: Sex was the key variable of interest. The remaining covariates were not discussed in the paper.
Abbreviation: hoh = head of household, SD = standard deviation.

Table S4: Sensitivity analysis for individual enrolment (outcome 1)

	Pooled			
	AOR	95% CI		p
sex ind				
...male	1			
...female	1.177	0.921	1.505	0.193
sex age				
...0-14	1			
...15-49	1.142	1.003	1.301	0.046
...50+	1.173	0.994	1.383	0.058
sex # age categories				
... female # 15-49	1.026	0.889	1.183	0.730
... female # 50+	1.069	0.898	1.272	0.453
relationship to hoh				
... hoh	1			
... spouse	0.897	0.589	1.366	0.614
... child	0.887	0.807	0.975	0.013
... others	0.611	0.530	0.705	0.000
sex # relationship				
... female # spouse	1.005	0.614	1.644	0.985
... female # child	0.787	0.625	0.992	0.042
... female # others	0.832	0.660	1.048	0.119
caste				
...No/ other/ general caste	1			
...scheduled caste/ tribe	1.307	1.117	1.529	0.001
...other backward class	1.185	1.013	1.386	0.034
religion				
...hindu	1			
...muslim	0.884	0.759	1.029	0.112
...christian/ other	0.618	0.466	0.820	0.001
education ind				
...no education	1			
...up to 7 years	1.012	0.935	1.097	0.762
...8 years and above	0.912	0.827	1.006	0.067
occupation ind				
...farming	1			
...labourer/ daily wages	0.904	0.805	1.014	0.086
...other occup	0.762	0.660	0.880	0.000
...student	1.060	0.923	1.217	0.408
...housewife	0.861	0.759	0.977	0.021
...not employed	0.912	0.793	1.049	0.198
...not yet in school	0.423	0.352	0.510	0.000
below poverty line				
...yes	1			
...no	0.727	0.648	0.815	0.000
household size	0.872	0.848	0.896	0.000
wealth index				
...lowest	1			
...second	1.128	0.959	1.326	0.146
...middle	1.291	1.097	1.518	0.002
...fourth	1.184	1.005	1.395	0.044
...highest	1.147	0.973	1.352	0.103
state (only for pooled sample)				
...Bihar	1			
...*Uttarakhand*	-			
...Uttar Pradesh	0.346	0.283	0.422	0.000
...West Bengal	0.761	0.619	0.935	0.009
...Gujarat	0.899	0.758	1.066	0.219
...Kerala	5.459	4.490	6.636	0.000
...Mizoram	7.862	5.532	11.175	0.000
...Tripura	3.662	3.055	4.389	0.000

Note: Sex was the key variable of interest. The remaining covariates were not discussed in the paper.
Baselines for the interaction *sex* and *age categories are* : male#0-14, male#15-49, male#50+, female#0-14.
Baselines for the interaction *sex* and *relationship to the head of household* : male#hoh, male#spouse, male#child, male#others, female#hoh.
Abbreviations: ind = individual, hoh = head of household, AOR = adjusted odds ratio, CI = confidence interval, p = p-value.

Table S5: Sensitivity analysis for household enrolment (outcome 2)

	Pooled			
	AOR	95% CI		p
sex hoh				
...male	1			
...female	1.283	1.105	1.491	0.001
age hoh				
...0-14	-			
...15-49	1			
...50+	0.860	0.744	0.995	0.043
caste				
...No/ other/ general caste	1			
...scheduled caste/ tribe	1.524	1.109	2.094	0.009
...other backward class	1.354	0.986	1.858	0.061
religion				
...hindu	1			
...muslim	0.969	0.573	1.639	0.907
...christian/ other	0.743	0.434	1.273	0.279
education hoh				
...no education	1			
...up to 7 years	0.987	0.891	1.094	0.809
...8 years and above	0.963	0.828	1.119	0.620
occupation hoh				
...farming	1			
...labourer/ daily wages	0.946	0.746	1.199	0.645
...other occup	0.853	0.692	1.052	0.138
...student	0.894	0.120	6.665	0.913
...housewife	0.990	0.689	1.422	0.955
...not employed	1.009	0.793	1.283	0.943
...not yet in schoool	-			
below poverty line				
...yes	1			
...no	0.619	0.285	1.346	0.226
household size	1.031	0.993	1.070	0.110
wealth index				
...lowest	1			
...second	1.172	0.888	1.546	0.262
...middle	1.363	1.089	1.705	0.007
...fourth	1.287	1.019	1.626	0.034
...highest	1.247	1.028	1.513	0.025
state (only for pooled sample)				
...Bihar	1			
...Uttarakhand	-			
...Uttar Pradesh	0.300	0.121	0.742	0.009
...West Bengal	0.828	0.396	1.731	0.615
...Gujarat	0.821	0.467	1.445	0.494
...Kerala	8.020	2.736	23.506	0.000
...Mizoram	7.190	3.479	14.858	0.000
...Tripura	6.169	2.733	13.925	0.000

Note: Sex was the key variable of interest. The remaining covariates were not discussed in the paper.
Abbreviations: hoh = head of household, AOR = adjusted odds ratio, CI = confidence interval, p = p-value.

Table S6: Results of the multivariate logistic regression for individual enrolment (outcome 1)

	Pooled				Bihar				Uttarakhand				Uttar Pradesh				West Bengal				Gujarat				Kerala				Mizoram				Tripura			
	AOR	95% CI		p	AOR	95% CI		p	AOR	95% CI		p	AOR	95% CI		p	AOR	95% CI		p	AOR	95% CI		p	AOR	95% CI		p	AOR	95% CI		p	AOR	95% CI		p
sex ind																																				
...male	1				1				1				1				1				1				1				1				1			
...female	1.265	1.003	1.595	0.048	1.289	0.670	2.470	0.445	2.664	1.315	5.397	0.007	0.793	0.354	1.772	0.571	0.964	0.359	2.591	0.943	0.990	0.484	2.027	0.978	1.246	0.648	2.399	0.510	0.978	0.496	1.929	0.950	1.146	0.547	2.400	0.717
age ind																																				
...0-14	1				1				1				1				1				1				1				1				1			
...15-49	1.139	1.007	1.289	0.038	0.900	0.660	1.230	0.511	1.197	0.808	1.772	0.369	0.991	0.717	1.371	0.957	1.064	0.645	1.755	0.808	1.518	1.113	2.071	0.008	1.373	0.914	2.061	0.130	1.048	0.703	1.561	0.819	1.094	0.746	1.603	0.646
...50+	1.173	1.003	1.372	0.046	0.836	0.550	1.270	0.406	1.234	0.725	2.101	0.438	0.883	0.563	1.386	0.590	0.936	0.509	1.721	0.831	1.537	1.046	2.258	0.029	2.130	1.298	3.496	0.000	0.978	0.566	1.691	0.937	1.218	0.725	2.045	0.456
sex # age categories																																				
... female # 15-49	0.996	0.870	1.140	0.952	1.178	0.800	1.720	0.400	0.685	0.448	1.047	0.080	1.044	0.712	1.531	0.825	1.099	0.651	1.857	0.723	0.801	0.555	1.157	0.237	1.022	0.652	1.604	0.920	1.178	0.798	1.740	0.409	0.836	0.562	1.244	0.377
... female # 50+	1.018	0.864	1.200	0.832	1.082	0.690	1.690	0.728	0.586	0.350	0.981	0.042	1.052	0.651	1.698	0.837	1.096	0.576	2.086	0.781	1.045	0.676	1.614	0.844	1.021	0.602	1.731	0.940	1.285	0.780	2.116	0.325	0.786	0.439	1.409	0.419
relationship to hoh																																				
... hoh	1				1				1				1				1				1				1				1				1			
... spouse	0.923	0.617	1.382	0.697	1.168	0.370	3.690	0.791	1.574	0.430	5.759	0.493	0.398	0.105	1.511	0.176	0.442	0.152	1.287	0.134	1.224	0.520	2.883	0.644	0.440	0.083	2.324	0.330	0.901	0.507	1.603	0.724	0.944	0.285	3.131	0.925
... child	0.885	0.809	0.967	0.007	0.612	0.470	0.800	0.000	0.836	0.636	1.098	0.197	0.891	0.680	1.168	0.404	1.016	0.747	1.382	0.920	1.008	0.814	1.250	0.940	1.097	0.820	1.466	0.530	0.836	0.611	1.144	0.262	0.606	0.449	0.818	0.001
... others	0.621	0.541	0.713	0.000	0.280	0.170	0.450	0.000	0.720	0.424	1.222	0.223	0.709	0.454	1.109	0.132	0.916	0.543	1.546	0.744	0.805	0.627	1.034	0.089	0.650	0.436	0.969	0.030	0.472	0.294	0.758	0.002	0.445	0.288	0.687	0.000
sex # relationship																																				
... female # spouse	0.930	0.582	1.485	0.761	0.638	0.170	2.360	0.501	0.357	0.085	1.497	0.159	2.747	0.555	13.583	0.215	1.642	0.395	6.833	0.495	1.126	0.339	3.741	0.846	1.982	0.319	12.332	0.460	1				1.543	0.383	6.211	0.542
... female # child	0.763	0.614	0.949	0.015	0.642	0.340	1.200	0.166	0.546	0.286	1.042	0.067	1.228	0.574	2.628	0.596	0.867	0.333	2.257	0.769	1.047	0.534	2.056	0.893	0.691	0.374	1.279	0.240	0.963	0.505	1.836	0.909	0.867	0.435	1.730	0.685
... female # others	0.800	0.642	0.998	0.048	0.718	0.380	1.360	0.312	0.530	0.253	1.111	0.093	1.009	0.468	2.174	0.982	0.540	0.212	1.378	0.197	1.039	0.545	1.979	0.908	0.721	0.386	1.347	0.310	1.406	0.702	2.817	0.336	1.091	0.516	2.306	0.819
caste																																				
...No/ other/ general caste	1				1				1				1				1				1				1				1				1			
...scheduled caste/ tribe	1.314	1.131	1.528	0.000	1.069	0.730	1.560	0.727	1.499	0.851	2.640	0.161	0.650	0.331	1.276	0.211	2.364	1.516	3.687	0.000	0.486	0.256	0.922	0.027	1.562	1.028	2.373	0.040	0.851	0.170	4.263	0.845	0.866	0.584	1.284	0.473
...other backward class	1.192	1.027	1.383	0.020	0.842	0.620	1.150	0.281	1.334	0.769	2.314	0.305	0.782	0.398	1.537	0.475	1.837	0.990	3.407	0.054	0.482	0.262	0.887	0.019	1.433	0.978	2.098	0.060	1				0.789	0.507	1.229	0.295
religion																																				
...hindu	1				1				1				1				1				1				1				1				1			
...muslim	0.867	0.753	0.998	0.046	0.994	0.720	1.370	0.971	0.725	0.480	1.094	0.126	1.031	0.506	2.100	0.934	0.742	0.510	1.079	0.118	0.398	0.132	1.204	0.103	1.201	0.846	1.706	0.310	1				0.504	0.318	0.797	0.003
...christian/ other	0.699	0.518	0.942	0.019	1				2.240	0.919	5.457	0.076	1				1				1.001	0.324	3.098	0.998	0.728	0.461	1.150	0.170	1.029	0.227	4.671	0.971	0.532	0.333	0.850	0.008
education ind																																				
...no education	1				1				1				1				1				1				1				1				1			
...up to 7 years	1.020	0.946	1.100	0.603	1.101	0.910	1.330	0.321	1.045	0.814	1.340	0.732	0.887	0.700	1.124	0.321	0.938	0.728	1.207	0.618	0.968	0.810	1.157	0.720	1.117	0.767	1.625	0.560	1.180	0.852	1.634	0.319	0.747	0.582	0.959	0.022
...8 years and above	0.942	0.859	1.033	0.203	1.004	0.780	1.300	0.975	1.156	0.880	1.518	0.297	0.981	0.756	1.274	0.886	0.793	0.578	1.089	0.152	0.972	0.775	1.220	0.809	0.878	0.593	1.299	0.510	1.098	0.758	1.592	0.620	0.725	0.537	0.979	0.036
occupation ind																																				
...farming	1				1				1				1				1				1				1				1				1			
...labourer/ daily wages	0.927	0.832	1.033	0.170	1.004	0.740	1.360	0.979	1.862	1.120	3.098	0.017	0.619	0.438	0.874	0.006	1.125	0.809	1.564	0.485	0.924	0.690	1.239	0.598	1.151	0.732	1.808	0.540	1.175	0.782	1.764	0.438	0.921	0.631	1.344	0.669
...other occupation	0.774	0.675	0.887	0.000	0.973	0.680	1.390	0.882	1.496	0.852	2.627	0.161	0.484	0.321	0.730	0.001	1.569	0.957	2.573	0.074	0.778	0.524	1.155	0.213	0.983	0.619	1.560	0.940	0.673	0.458	0.989	0.044	0.548	0.357	0.842	0.006
...student	1.056	0.926	1.205	0.413	1.503	1.030	2.200	0.036	1.864	1.114	3.121	0.018	0.815	0.568	1.169	0.265	0.976	0.587	1.622	0.924	0.946	0.710	1.262	0.707	1.529	0.899	2.601	0.120	1.274	0.865	1.874	0.220	1.128	0.724	1.757	0.594
...housewife	0.886	0.785	1.001	0.052	1.021	0.680	1.540	0.921	1.887	1.080	3.298	0.026	0.850	0.593	1.219	0.376	1.625	1.058	2.497	0.027	0.863	0.672	1.109	0.250	1.144	0.704	1.858	0.590	0.981	0.679	1.417	0.919	0.500	0.309	0.808	0.005
...not employed	0.928	0.811	1.061	0.274	1.191	0.800	1.770	0.388	2.034	1.106	3.740	0.022	0.808	0.518	1.261	0.347	1.566	0.949	2.584	0.079	0.797	0.587	1.082	0.146	1.405	0.865	2.280	0.170	1.051	0.730	1.513	0.789	0.639	0.413	0.989	0.044
...not yet in school	0.427	0.357	0.511	0.000	0.666	0.430	1.030	0.066	0.754	0.356	1.600	0.462	0.347	0.211	0.574	0.000	0.555	0.262	1.178	0.125	0.363	0.230	0.574	0.000	0.340	0.166	0.697	0.003	0.724	0.435	1.205	0.214	0.208	0.112	0.385	0.000
below poverty line																																				
...yes	1				1				1				1				1				1				1				1				1			
...no	0.739	0.663	0.824	0.000	0.751	0.540	1.040	0.085	0.835	0.597	1.169	0.295	1.664	1.212	2.286	0.002	0.072	0.051	0.100	0.000	1.896	1.491	2.410	0.000	1.104	0.785	1.553	0.570	0.614	0.436	0.865	0.005	0.683	0.505	0.924	0.013
household size	0.883	0.860	0.907	0.000	0.932	0.880	0.990	0.018	0.979	0.901	1.064	0.617	0.875	0.826	0.927	0.000	0.939	0.841	1.049	0.265	0.913	0.860	0.970	0.003	0.836	0.749	0.933	0.001	0.614	0.542	0.695	0.000	0.836	0.769	0.909	0.000
wealth index																																				
...lowest	1				1				1				1				1				1				1				1				1			
...second	1.054	0.902	1.233	0.506	1.369	0.940	1.990	0.101	0.538	0.324	0.894	0.017	0.679	0.418	1.103	0.118	1.423	0.870	2.325	0.160	0.929	0.611	1.411	0.728	0.991	0.608	1.614	0.970	2.197	1.355	3.562	0.001	1.011	0.651	1.569	0.961
...middle	1.173	1.004	1.371	0.044	1.335	0.920	1.950	0.132	0.477	0.284	0.803	0.005	0.650	0.402	1.051	0.079	1.744	1.050	2.896	0.032	1.645	1.100	2.460	0.015	1.250	0.777	2.011	0.360	1.926	1.172	3.166	0.010	1.055	0.691	1.610	0.804
...fourth	1.102	0.941	1.290	0.230	1.053	0.720	1.540	0.790	0.586	0.350	0.983	0.043	0.916	0.573	1.464	0.715	0.956	0.557	1.641	0.871	1.732	1.153	2.602	0.008	1.052	0.657	1.686	0.830	1.643	1.013	2.663	0.044	1.151	0.749	1.768	0.522
...highest	1.084	0.925	1.271	0.320	1.332	0.910	1.940	0.137	0.694	0.415	1.161	0.164	0.991	0.623	1.577	0.971	0.879	0.505	1.529	0.648	1.561	1.044	2.333	0.030	0.927	0.565	1.522	0.770	1.287	0.790	2.097	0.311	1.020	0.676	1.537	0.926
state (only for pooled sample)																																				
...Bihar	1																																			
...Uttarakhand	0.288	0.235	0.353	0.000																																
...Uttar Pradesh	0.343	0.282	0.418	0.000																																
...West Bengal	0.778	0.634	0.954	0.016																																
...Gujarat	0.895	0.756	1.060	0.198																																
...Kerala	5.348	4.410	6.486	0.000																																
...Mizoram	6.900	4.805	9.908	0.000																																
...Tripura	3.638	3.041	4.353	0.000																																

Note: sex was the key variable of interest. The remaining covariates were not discussed in the paper.
Baselines for the interaction *sex* and *age categories* : male#0-14, male#15-49, male#50+, female#0-14;
Baselines for the interaction *sex* and *relationship to the head of household* : male#hoh, male#spouse, male#child, male#others, female#hoh.
Abbreviations: ind = individual, AOR = adjusted odds ratio, CI = confidence interval, p = p-value.

Table S7: Results of the multivariate logistic regression for household enrolment (outcome 2)

	Pooled				Bihar				Uttarakhand			
	AOR	95% CI		p	AOR	95% CI		p	AOR	95% CI		p
sex hoh												
...male	1				1				1			
...female	1.358	1.142	1.616	0.001	1.460	0.968	2.202	0.071	2.507	1.142	5.503	0.022
age hoh												
...0-14	-				-				-			
...15-49	1				1				1			
...50+	0.872	0.759	1.001	0.051	0.776	0.450	1.337	0.361	0.967	0.575	1.626	0.900
caste												
...No/ other/ general caste	1				1				1			
...scheduled caste/ tribe	1.513	1.138	2.010	0.004	1.100	1.076	1.123	0.000	1.666	1.408	1.970	0.000
...other backward class	1.355	1.009	1.821	0.044	0.840	0.699	1.008	0.061	1.499	0.797	2.820	0.209
religion												
...hindu	1				1				1			
...muslim	0.926	0.601	1.428	0.729	1.133	0.937	1.370	0.197	0.785	0.654	0.942	0.009
...christian/ other	0.850	0.517	1.398	0.522	1				1.744	1.130	2.691	0.012
education hoh												
...no education	1				1				1			
...up to 7 years	1.025	0.909	1.156	0.684	1.079	0.775	1.502	0.652	1.334	0.795	2.240	0.275
...8 years and above	0.998	0.854	1.167	0.983	1.067	1.025	1.111	0.002	1.301	0.735	2.304	0.367
occupation hoh												
...farming	1				1				1			
...labourer/ daily wages	0.978	0.783	1.222	0.846	1.415	0.706	2.837	0.328	1.573	1.045	2.368	0.030
...other occupation	0.865	0.709	1.054	0.150	1.480	0.670	3.273	0.332	1.213	0.963	1.529	0.101
...student	0.666	0.114	3.877	0.651	-				1			
...housewife	0.932	0.670	1.296	0.675	1.148	0.322	4.096	0.831	0.701	0.295	1.668	0.422
...not employed	1.054	0.841	1.322	0.648	1.947	0.815	4.650	0.134	1.978	0.788	4.964	0.146
...not yet in schoool	-				-				-			
below poverty line												
...yes	1				1				1			
...no	0.641	0.320	1.283	0.209	0.738	0.428	1.272	0.274	0.878	0.375	2.055	0.764
household size	1.035	1.001	1.071	0.042	1.049	0.980	1.122	0.168	1.079	1.026	1.134	0.003
wealth index												
...lowest	1				1				1			
...second	1.095	0.837	1.432	0.508	1.549	1.019	2.353	0.041	0.583	0.485	0.701	0.000
...middle	1.229	0.966	1.564	0.093	1.381	1.061	1.798	0.016	0.497	0.339	0.727	0.000
...fourth	1.177	0.917	1.509	0.200	1.297	1.064	1.582	0.010	0.578	0.541	0.618	0.000
...highest	1.197	0.987	1.451	0.067	1.544	1.090	2.187	0.014	0.815	0.654	1.015	0.067
state (only for pooled sample)												
...Bihar	1											
...Uttarakhand	0.255	0.160	0.408	0.000								
...Uttar Pradesh	0.300	0.123	0.735	0.008								
...West Bengal	0.839	0.421	1.671	0.617								
...Gujarat	0.813	0.471	1.403	0.456								
...Kerala	7.641	2.692	21.690	0.000								
...Mizoram	6.079	3.124	11.829	0.000								
...Tripura	6.023	2.709	13.390	0.000								

	Uttar Pradesh				West Bengal				Gujarat			
	AOR	95% CI		p	AOR	95% CI		p	AOR	95% CI		p
sex hoh												
...male	1				1				1			
...female	0.862	0.813	0.914	0.000	1.278	0.772	2.116	0.340	0.958	0.705	1.302	0.783
age hoh												
...0-14	-				-				-			
...15-49	1				1				1			
...50+	0.696	0.692	0.699	0.002	0.780	0.482	1.264	0.313	1.027	0.722	1.462	0.881
caste												
...No/ other/ general caste	1				1				1			
...scheduled caste/ tribe	0.631	0.369	1.082	0.094	2.150	1.759	2.628	0.000	0.519	0.168	1.606	0.255
...other backward class	0.808	0.426	1.534	0.515	1.712	0.596	4.918	0.318	0.471	0.174	1.276	0.139
religion												
...hindu	1				1				1			
...muslim	0.964	0.260	3.574	0.956	0.681	0.290	1.602	0.379	0.521	0.269	1.012	0.054
...christian/ other	1				1				2.455	0.909	6.632	0.077
education hoh												
...no education	1				1				1			
...up to 7 years	0.688	0.593	0.799	0.000	1.024	0.793	1.321	0.858	1.000	0.960	1.043	0.983
...8 years and above	1.058	0.891	1.258	0.519	1.112	0.709	1.743	0.645	1.055	0.793	1.403	0.713
occupation hoh												
...farming	1				1				1			
...labourer/ daily wages	0.588	0.527	0.656	0.000	1.220	0.785	1.897	0.376	0.928	0.675	1.276	0.647
...other occupation	0.915	0.679	1.234	0.561	1.198	1.163	1.234	0.000	0.624	0.423	0.920	0.017
...student	1				1				1			
...housewife	1.381	0.435	4.380	0.583	2.008	0.509	7.921	0.319	2.438	0.858	6.930	0.095
...not employed	0.628	0.371	1.064	0.084	0.783	0.326	1.884	0.585	1.103	0.483	2.521	0.816
...not yet in schoool	-				-				-			
below poverty line												
...yes	1				1				1			
...no	1.558	1.022	2.376	0.039	0.052	0.012	0.223	0.000	1.844	0.884	3.846	0.103
household size	1.012	0.976	1.049	0.506	1.151	1.024	1.293	0.018	1.061	0.982	1.145	0.132
wealth index												
...lowest	1				1				1			
...second	0.750	0.563	0.999	0.049	1.360	0.346	5.345	0.660	1.023	0.817	1.282	0.841
...middle	0.782	0.686	0.891	0.000	1.349	0.362	5.029	0.655	1.767	1.330	2.348	0.000
...fourth	1.092	1.088	1.096		0.793	0.206	3.050	0.736	1.946	1.389	2.725	0.000
...highest	1.139	0.724	1.794	0.573	0.815	0.319	2.082	0.669	1.683	1.621	1.747	0.000

	Kerala				Mizoram				Tripura			
	AOR	95% CI		p	AOR	95% CI		p	AOR	95% CI		p
sex hoh												
...male	1				1				1			
...female	1.056	0.791	1.409	0.712	1.652	0.955	2.857	0.072	1.126	0.493	2.568	0.778
age hoh												
...0-14	-				-				-			
...15-49	1				1				1			
...50+	1.312	0.768	2.241	0.321	0.701	0.438	1.121	0.138	1.016	0.940	1.099	0.687
caste												
...No/ other/ general caste	1				1				1			
...scheduled caste/ tribe	1.831	0.472	7.102	0.382	1				0.961	0.737	1.255	0.772
...other backward class	1.895	0.905	3.968	0.090	1				0.799	0.760	0.839	0.000
religion												
...hindu	1				1				1			
...muslim	1.552	1.133	2.127	0.006	1				0.385	0.196	0.757	0.006
...christian/ other	0.742	0.301	1.830	0.517	0.803	0.147	4.390	0.800	0.767	0.756	0.778	0.000
education hoh												
...no education	1				1				1			
...up to 7 years	0.825	0.813	0.837	0.000	1.164	0.418	3.237	0.772	0.804	0.570	1.136	0.216
...8 years and above	0.439	0.428	0.451		0.964	0.237	3.919	0.959	0.885	0.848	0.924	0.000
occupation hoh												
...farming	1				1				1			
...labourer/ daily wages	1.405	0.383	5.159	0.609	1.433	0.660	3.108	0.363	1.104	0.734	1.659	0.635
...other occupation	1.162	0.996	1.356	0.057	0.684	0.477	0.981	0.039	0.546	0.316	0.943	0.030
...student	0.287	0.030	2.741	0.279	1				-			
...housewife	1.683	0.565	5.014	0.350	0.655	0.266	1.616	0.359	0.534	0.056	5.109	0.586
...not employed	1.601	0.778	3.293	0.201	0.918	0.684	1.232	0.569	0.827	0.519	1.315	0.422
...not yet in schoool	-				-				-			
below poverty line												
...yes	1				1				1			
...no	1.657	1.053	2.609	0.029	0.529	0.468	0.599	0.000	0.537	0.480	0.600	0.000
household size	1.023	1.020	1.025	0.000	0.803	0.695	0.927	0.003	1.075	0.981	1.178	0.119
wealth index												
...lowest	1				1				1			
...second	1.385	0.619	3.096	0.428	2.488	0.776	7.978	0.125	0.886	0.849	0.925	0.000
...middle	2.070	1.773	2.418	0.000	2.311	1.716	3.113	0.000	1.122	0.458	2.748	0.801
...fourth	1.469	0.800	2.697	0.214	2.023	1.798	2.277	0.000	1.168	0.706	1.935	0.545
...highest	1.228	0.686	2.199	0.489	1.556	0.743	3.257	0.241	1.188	1.077	1.310	0.001

Note: Sex was the key variable of interest. The remaining covariates were not discussed in the paper.
Abbreviations: hoh = head of household, AOR = adjusted odds ratio, CI = confidence interval, p = p-value.

Table S8: Results of the multivariate logistic regression for complete household enrolment (outcome 3)

	Pooled				Bihar				Uttarakhand				Uttar Pradesh			
	AOR	95% CI		p	AOR	95% CI		p	AOR	95% CI		p	AOR	95% CI		p
sex hoh																
...male	1				1				1				1			
...female	0.826	0.603	1.130	0.232	0.893	0.120	6.640	0.912	0.419	0.117	1.505	0.183	1.739	0.145	20.916	0.663
age hoh																
...0-14	-				-				-				-			
...15-49	1				1				1				1			
...50+	0.988	0.839	1.164	0.890	1.277	0.851	1.917	0.237	0.429	0.251	0.733	0.002	0.775	0.364	1.651	0.509
caste																
...No/ other/ general caste	1				1				1				1			
...scheduled caste/ tribe	0.899	0.754	1.073	0.239	0.738	0.703	0.775	0.000	0.678	0.162	2.846	0.596	0.513	0.190	1.384	0.187
...other backward class	0.763	0.650	0.896	0.001	0.559	0.478	0.654	0.000	0.701	0.229	2.144	0.534	0.351	0.076	1.627	0.181
religion																
...hindu	1				1				1				1			
...muslim	0.815	0.657	1.010	0.061	0.661	0.460	0.949	0.025	0.412	0.262	0.647	0.000	1.308	0.401	4.259	0.656
...christian/ other	0.587	0.443	0.778	0.000					2.081	0.018	243.392	0.763				
education hoh																
...no education	1				1				1				1			
...up to 7 years	0.932	0.807	1.078	0.344	1.075	0.843	1.371	0.560	0.397	0.188	0.839	0.016	1.014	0.374	2.750	0.978
...8 years and above	1.000	0.827	1.210	0.999	1.973	1.380	2.821	0.000	0.574	0.388	0.849	0.005	0.681	0.625	0.741	0.000
occupation hoh																
...farming	1				1				1				1			
...labourer/ daily wages	1.104	0.901	1.352	0.340	2.140	1.526	3.000	0.000	0.953	0.196	4.622	0.952	0.753	0.560	1.012	0.060
...other occupation	1.095	0.774	1.551	0.608	2.807	1.014	7.772	0.047	0.745	0.059	9.437	0.820	0.548	0.543	0.553	
...student	0.483	0.030	7.691	0.606	-				-				-			
...housewife	1.346	0.760	2.386	0.308	2.125	0.166	27.177	0.562	2.573	0.331	20.019	0.367	0.294	0.037	2.311	0.245
...not employed	0.821	0.548	1.232	0.341	1.058	0.643	1.742	0.824	1.860	0.198	17.428	0.587	0.145	0.035	0.599	0.008
...not yet in schoool	-				-				-				-			
below poverty line																
...yes	1				1				1				1			
...no	0.984	0.811	1.194	0.871	1.290	0.795	2.094	0.303	0.860	0.711	1.040	0.121	0.942	0.548	1.619	0.828
household size	0.847	0.782	0.918	0.000	0.989	0.894	1.095	0.836	0.937	0.811	1.084	0.382	0.798	0.691	0.922	0.002
wealth index																
...lowest	1				1				1				1			
...second	0.807	0.521	1.251	0.337	0.718	0.612	0.842	0.000	0.267	0.177	0.404	0.000	0.304	0.132	0.703	0.005
...middle	0.955	0.688	1.327	0.785	1.496	0.834	2.684	0.177	0.287	0.077	1.074	0.064	0.575	0.266	1.242	0.159
...fourth	0.726	0.512	1.029	0.072	0.596	0.437	0.813	0.001	0.602	0.110	3.287	0.558	0.488	0.045	5.307	0.556
...highest	0.675	0.468	0.975	0.036	0.709	0.252	2.000	0.516	0.225	0.101	0.499	0.000	0.364	0.030	4.420	0.427
state (only for pooled sample)																
...Bihar	1															
...Uttarakhand	1.539	1.197	1.979	0.001												
...Uttar Pradesh	1.131	0.534	2.397	0.747												
...West Bengal	0.897	0.635	1.265	0.535												
...Gujarat	1.499	0.888	2.531	0.130												
...Kerala	1.551	1.146	2.099	0.004												
...Mizoram	6.942	4.595	10.488	0.000												
...Tripura	0.839	0.616	1.143	0.266												

	West Bengal				Gujarat				Kerala				Mizoram				Tripura			
	AOR	95% CI		p	AOR	95% CI		p	AOR	95% CI		p	AOR	95% CI		p	AOR	95% CI		p
sex hoh																				
...male	1				1				1				1				1			
...female	0.700	0.297	1.652	0.415	0.803	0.500	1.289	0.363	0.626	0.300	1.308	0.213	0.704	0.178	2.784	0.617	1.049	0.907	1.212	0.522
age hoh																				
...0-14	-				-				-				-				-			
...15-49	1				1				1				1				1			
...50+	0.990	0.289	3.388	0.987	0.828	0.465	1.475	0.522	0.884	0.695	1.124	0.314	1.000	0.750	1.333	0.999	1.120	0.817	1.535	0.483
caste																				
...No/ other/ general caste	1				1				1				1				1			
...scheduled caste/ tribe	1.738	0.941	3.211	0.077	0.932	0.606	1.434	0.749	1.034	0.974	1.098	0.278	2.235	0.040	125.215	0.695	0.729	0.554	0.960	0.024
...other backward class	1.550	0.503	4.780	0.445	0.968	0.627	1.495	0.882	0.760	0.691	0.837	0.000	1				0.674	0.390	1.165	0.158
religion																				
...hindu	1				1				1				1				1			
...muslim	1.336	0.710	2.511	0.369	0.041	0.004	0.465	0.010	1.105	0.902	1.353	0.336	1				0.772	0.517	1.153	0.207
...christian/ other	1				0.140	0.003	5.953	0.304	0.761	0.605	0.958	0.020	3.972	0.072	219.647	0.501	0.574	0.467	0.706	0.000
education hoh																				
...no education	1				1				1				1				1			
...up to 7 years	0.742	0.601	0.916	0.006	1.080	0.604	1.930	0.795	1.042	0.876	1.240	0.644	0.154	0.066	0.358	0.000	0.916	0.762	1.102	0.354
...8 years and above	0.970	0.574	1.638	0.908	1.299	1.175	1.436	0.000	1.394	1.343	1.446	0.000	0.099	0.036	0.267	0.000	0.821	0.586	1.149	0.249
occupation hoh																				
...farming	1				1				1				1				1			
...labourer/ daily wages	1.028	0.360	2.934	0.959	1.606	1.253	2.058	0.000	0.954	0.700	1.300	0.765	0.876	0.260	2.945	0.830	0.922	0.752	1.132	0.439
...other occupation	1.108	0.279	4.404	0.884	0.756	0.296	1.932	0.559	1.299	0.909	1.856	0.151	1.022	0.422	2.476	0.961	0.768	0.223	2.644	0.675
...student	1				1				1				1				-			
...housewife	0.614	0.218	1.727	0.355	1				2.565	1.483	4.437	0.001	1				0.558	0.539	0.576	0.000
...not employed	2.049	0.024	173.322	0.751	0.708	0.183	2.732	0.616	1.183	0.844	1.658	0.328	0.716	0.520	0.987	0.041	0.521	0.155	1.756	0.293
...not yet in schoool	-				-				-				-				-			
below poverty line																				
...yes	1				1				1				1				1			
...no	0.841	0.400	1.770	0.648	1.649	1.106	2.459	0.014	0.717	0.696	0.738	0.000	1.062	0.259	4.353	0.934	0.925	0.848	1.008	0.077
household size	0.633	0.596	0.672	0.000	0.894	0.888	0.900	0.000	0.757	0.601	0.954	0.018	0.805	0.648	0.999	0.049	0.796	0.676	0.938	0.006
wealth index																				
...lowest	1				1				1				1				1			
...second	1.090	0.572	2.079	0.793	0.413	0.392	0.435	0.000	0.462	0.258	0.828	0.009	1.717	1.596	1.847	0.000	1.713	0.476	6.160	0.410
...middle	2.538	1.551	4.154	0.000	0.399	0.181	0.880	0.023	0.634	0.343	1.172	0.146	1.049	0.998	1.102	0.061	1.330	0.740	2.389	0.340
...fourth	2.312	1.490	3.588	0.000	0.402	0.247	0.653	0.000	0.366	0.248	0.540	0.000	0.997	0.735	1.351	0.983	1.182	0.690	2.023	0.543
...highest	1.253	0.336	4.671	0.737	0.461	0.394	0.541	0.000	0.390	0.280	0.542	0.000	1.169	0.195	7.012	0.865	1.017	0.478	2.161	0.966

Note: Sex was the key variable of interest. The remaining covariates were not discussed in the paper.
Abbreviations: hoh = head of household, AOR = adjusted odds ratio, CI = confidence interval, p = p-value.
